# Current and Future Technologies for the Detection of Antibiotic-Resistant Bacteria

**DOI:** 10.3390/diagnostics13203246

**Published:** 2023-10-18

**Authors:** Dina Yamin, Vuk Uskoković, Abubakar Muhammad Wakil, Mohammed Dauda Goni, Shazana Hilda Shamsuddin, Fatin Hamimi Mustafa, Wadha A. Alfouzan, Mohammed Alissa, Amer Alshengeti, Rana H. Almaghrabi, Mona A. Al Fares, Mohammed Garout, Nawal A. Al Kaabi, Ahmad A. Alshehri, Hamza M. Ali, Ali A. Rabaan, Fatimah Abdullah Aldubisi, Chan Yean Yean, Nik Yusnoraini Yusof

**Affiliations:** 1 Al-Karak Public Hospital, Karak 61210, Jordan; dr.dinayamin@gmail.com; 2 Institute for Research in Molecular Medicine, University Sains Malaysia, Health Campus, Kubang Kerian 16150, Kelantan, Malaysia; 3 Department of Veterinary Clinical Studies, Faculty of Veterinary Medicine, University Malaysia Kelantan, Kota Bharu 16100, Kelantan, Malaysia; abubakar.mw@umk.edu.my; 4 TardigradeNano LLC., Irvine, CA 92604, USA; vuk.uskokovic@tardigradenano.com; 5 Department of Mechanical Engineering, San Diego State University, San Diego, CA 92182, USA; 6 Department of Veterinary Physiology and Biochemistry, Faculty of Veterinary Medicine, University of Maiduguri, Maiduguri 600104, Borno, Nigeria; 7 Public Health and Zoonoses Research Group, Faculty of Veterinary Medicine, University Malaysia Kelantan, Pengkalan Chepa 16100, Kelantan, Malaysia; dauda.g@umk.edu.my; 8 Department of Pathology, School of Medical Sciences, University Sains Malaysia, Health Campus, Kubang Kerian 16150, Kelantan, Malaysia; shazana.hilda@usm.my; 9 Department of Electronic & Computer Engineering, Faculty of Electrical Engineering, University Teknologi Malaysia, Johor Bharu 81310, Johor, Malaysia; fatinhamimi@utm.my; 10 Department of Microbiology, Faculty of Medicine, Kuwait University, Safat 13110, Kuwait; alfouzan.w@ku.edu.kw; 11 Microbiology Unit, Department of Laboratories, Farwania Hospital, Farwania 85000, Kuwait; 12 Department of Medical Laboratory Sciences, College of Applied Medical Sciences, Prince Sattam bin Abdulaziz University, Al-Kharj 11942, Saudi Arabia; m.alissa@psau.edu.sa; 13 Department of Pediatrics, College of Medicine, Taibah University, Al-Madinah 41491, Saudi Arabia; aalshengeti@dal.ca; 14 Department of Infection Prevention and Control, Prince Mohammad Bin Abdulaziz Hospital, National Guard Health Affairs, Al-Madinah 41491, Saudi Arabia; 15 Pediatric Department, Prince Sultan Medical Military City, Riyadh 12233, Saudi Arabia; ralmaghrabi@psmmc.med.sa; 16 College of Medicine, Alfaisal University, Riyadh 11533, Saudi Arabia; arabaan@gmail.com; 17 Department of Internal Medicine, King Abdulaziz University Hospital, Jeddah 21589, Saudi Arabia; maalfares@kau.edu.sa; 18 Department of Community Medicine and Health Care for Pilgrims, Faculty of Medicine, Umm Al-Qura University, Makkah 21955, Saudi Arabia; magarout@uqu.edu.sa; 19 College of Medicine and Health Science, Khalifa University, Abu Dhabi 127788, United Arab Emirates; alkaabin971@gmail.com; 20 Sheikh Khalifa Medical City, Abu Dhabi Health Services Company (SEHA), Abu Dhabi 51900, United Arab Emirates; 21 Department of Clinical Laboratory Sciences, Faculty of Applied Medical Sciences, Najran University, Najran 61441, Saudi Arabia; aaalshehri@nu.edu.sa; 22 Department of Medical Laboratories Technology, College of Applied Medical Sciences, Taibah University, Madinah 41411, Saudi Arabia; heid@taibahu.edu.sa; 23 Molecular Diagnostic Laboratory, Johns Hopkins Aramco Healthcare, Dhahran 31311, Saudi Arabia; 24 Department of Public Health and Nutrition, The University of Haripur, Haripur 22610, Pakistan; 25 Pediatrics Department, Riyadh Second Health Cluster, Riyadh 13245, Saudi Arabia; fdubisi@hotmail.com; 26 Department of Medical Microbiology & Parasitology, School of Medical Sciences, University Sains Malaysia, Kubang Kerian 16150, Kelantan, Malaysia

**Keywords:** antimicrobial resistance (AMR), antibiotic-resistant bacteria, PCR, biosensors, aptamers

## Abstract

Antibiotic resistance is a global public health concern, posing a significant threat to the effectiveness of antibiotics in treating bacterial infections. The accurate and timely detection of antibiotic-resistant bacteria is crucial for implementing appropriate treatment strategies and preventing the spread of resistant strains. This manuscript provides an overview of the current and emerging technologies used for the detection of antibiotic-resistant bacteria. We discuss traditional culture-based methods, molecular techniques, and innovative approaches, highlighting their advantages, limitations, and potential future applications. By understanding the strengths and limitations of these technologies, researchers and healthcare professionals can make informed decisions in combating antibiotic resistance and improving patient outcomes.

## 1. Introduction

### 1.1. Antibiotic Resistance: A Growing Global Health Challenge

The evolution and manufacture of antibiotics in the last century have been among the most outstanding achievements in the field of medicine [1]. Antimicrobials are being recognized as one of the most efficacious forms of chemotherapy in the annals of medical progress. It is unnecessary to mention the number of lives antibiotics have rescued and how greatly they helped to control infectious diseases, which were the primary reasons for human death and morbidity before the advent of antibiotics [2]. Today, antibiotics are either therapeutic or prophylactic drugs and are commonly utilized in medicine and agriculture. The first identified antibacterial chemical was penicillin [3], a β-lactam antibiotic. Soon after this remarkable discovery, antibiotics were employed to treat human illnesses, starting with sulfonamide and followed by the aminoglycosides streptomycin and streptothricin [1,4,5].

Antimicrobial resistance (AMR) refers to the capacity of pathogens and other microbes to withstand the influence of an antibiotic that was formerly vulnerable, thus allowing the organisms to prevail and thrive [6]. AMR is unavoidable because microorganisms adopt genetic changes to reduce the fatal effects of antibiotic therapy [7,8]. The natural history of genes responsible for antibiotic resistance traces the phylogenetic reconstruction. It has unveiled the enduring existence of genetic elements that provide resistance to various classes of antibiotics in natural environments, predating the advent of antibiotics [9,10]. Phylogenetic analysis based on the structure of serine and Metallo-β-lactamases, for instance, revealed that these primitive enzymes developed more than two billion years ago, with specific serine beta-lactamases found on plasmids for millions of years [11,12]. Prior to the general application of penicillin in medicine, data revealed that bacteria can degrade this compound through an enzymatic breakdown [2,13]. AMR was originally discovered in gonococci, streptococci, and staphylococci. In fact, following the commercially produced antibiotic, penicillin became available in 1941. It took only a year before penicillin-resistant *Staphylococcus aureus* developed [14]. Subsequently, a derivative of penicillin was launched into the marketplace in 1960 to address penicillin-resistant *S. aureus*, which also developed resistance to methicillin that same year [8,15].

AMR is raising a huge concern up to date due to its widespread use and the short duration of antibiotics to acquire resistance. Almost 70% of bacteria causing infections are resistant to at least one medication [16]. AMR is now considered one of the most critical risks to public health in health care facilities, as well as in food protection. According to the 2019 Antibiotic Resistance Threats Report by the Centers for Disease Control and Prevention (CDC), over 2.8 million cases of antibiotic-resistant infections occur annually in the United States, with over 35,000 fatalities [17]. Similarly, a survey carried out in India showed that an alarming rate of child mortality due to antibiotic-resistant bacterial infection occurs almost every 9 min and over 50,000 fatalities among infants as a result of sepsis due to germs becoming resistant to standard antibiotics [7]. The World Health Organization (WHO) reported in 2019 that AMR causes the deaths of 700,000 people worldwide each year, and by 2050, it will result in the deaths of 20 million, costing over $2.9 trillion in healthcare [8]. In addition, from 2015 to 2019, the European Antimicrobial Resistance Surveillance Network (EARS-Net) [18] revealed that the prevalence of AMR in the European Union is changing in relation to bacterial species, antibiotic class, and geographical location. According to the survey conducted, methicillin-resistant *Staphylococcus aureus* (MRSA) is continuously posing threats with as high as 74% of all *S. aureus* infections globally. *S. aureus* impacts approximately 120,000 persons in the US, resulting in nearly 20,000 deaths [19], and the number of deaths attributable solely to MRSA exceeded 100,000 in 2019 [15]. In its most recent Global Antimicrobial Surveillance System (GLASS) [20], the WHO demonstrated widespread AMR across 500,000 people with documented bacterial infections in 22 different countries. The most frequently encountered antibiotic-resistant bacteria included *Escherichia coli*, *S. aureus*, *Streptococcus pneumoniae*, and *Klebsiella pneumoniae* [15]. Ciprofloxacin, a commonly used antibiotic for urinary tract infections (UTIs), has shown varying levels of resistance across different bacterial strains. Resistance to *E. coli* ranged from 8.4% to 92.9%, while for *K. pneumoniae*, it ranged from 4.1% to 79.4%. Penicillin resistance reached 51% in multiple countries, as reported by the GLASS. In 2019, the GLASS collected data on MRSA bloodstream infections (BSIs) from 25 countries, zones, and regions, as well as *E. coli* bloodstream infection data from 49 countries. According to a study [21], MRSA had a median prevalence of 12.11%. Additionally, the study revealed a 36% resistance rate among *E. coli* to third-generation cephalosporins.

According to the findings presented by Migliori et al. [22], the data pertaining to the monitoring of drug resistance in patients with tuberculosis (TB) indicated that 3.5% of the current patients with TB and 18% with previous case of TB treatment globally are projected to develop specifically rifampicin-resistant (RR) TB or even a multidrug-resistant (MDR) [22]. In 2017, there was a notable global rise in the incidence of new cases of MDR/RR-TB recorded, which resulted in the deaths of 230,000 people that year [8,23].

### 1.2. Importance of Accurate Detection of Antibiotic-Resistant Bacteria

Healthcare-associated infections (HAIs) are the most frequent leading cause of clinical complications, affecting 5% to 10% of patients and increasing healthcare costs substantially, with expenses encompassing medical treatment, extended hospital stays, and legal fees associated with HAI-related litigations, totaling around USD 4 billion [24]. Moreover, the emergence of AMR bacteria in HAIs further complicates the financial burden. These multidrug-resistant organisms can necessitate specialized treatments and longer hospitalization, significantly escalating costs. Effective risk management in HAI prevention is crucial to curb both the incidence of HAIs and the spread of AMR. It involves strict adherence to infection control practices, prudent antibiotic use, and surveillance to detect and manage resistant pathogens, all of which are vital to mitigate the economic impact and enhance patient safety [24]. Action plans are being developed by the WHO and other organizations in this sector to enhance the understanding of AMR, reduce the prevalence of infectious diseases through the implementation of infection prevention measures, optimize antimicrobial treatments, create new drugs and treatment strategies, and improve the effectiveness of antimicrobial medicines. It should be noted that there has been significant progress in preventing AMR with these strategies. Although enormous efforts have been undertaken to address AMR, it remains an inescapable issue that diminishes the efficacy of antibiotics in the treatment of infections.

For infectious illnesses to be treated as effectively as possible, early infection diagnosis is essential. Despite significant advancements in medical technology, the detection and diagnosis of microbiological diseases often require several days to complete, leading to a prolonged turnaround time (TAT) [25,26,27]. For this reason, physicians are driven to initiate practically achievable antibiotic therapies, often broad-spectrum, prior to carrying a diagnosis. This technique may lead to adverse repercussions not only for the health of the patient but also in terms of exacerbating the developing AMR crisis. Thus, the demand for speedy, sensitive, and affordable kits for AMR diagnostics has become critical. The adoption of such avenues will greatly lower the TAT for antibiotic susceptibility determination, thereby allowing the selection of improved, target-specific medicines [25]. In short, diagnostic testing is considered a vital tool in any approach against AMR [28].

Generally, a clinical microbiology analysis focuses on phenotypic approaches to evaluate the antimicrobial susceptibility patterns of pathogens [29]. These methods are still relevant because of their benefits, such as being affordable and easy to carry out and having a clear criterion for interpretation. However, they are too cumbersome to provide rapid susceptibility data, thereby delaying the initiation of the therapy [30]. These restrictions have been discovered to have ramifications in patient management; for example, a delay in the commencement of the antibiotic therapy has been linked to increases in both mortality [31] and hospitalization time [32]. This has made it difficult to apply the back-end method of the antimicrobial stewardship program, which has proven gratifying outcomes in patient management and the battle against AMR [33]. To overcome the limitations of the phenotypic approach and enhance both patient care and the management of antibiotic resistance, continuous endeavors are underway to advance the development of rapid antimicrobial susceptibility testing (AST) technologies. These techniques are designed to expedite the identification of pathogenic organisms and assess their susceptibility to antimicrobial agents.

In clinical diagnostics, there are approximately five different methods to speed up AST: (I) bypassing traditional culture methods by directly detecting the pathogen or resistance mechanism in the initial sample; (II) eliminating the need for secondary culture-based susceptibility testing; (III) reducing the time required for analysis and improving the sensitivity for detecting the infectious agent; (IV) enhancing early identification of the pathogen in the initial stages of the disease and at lower microbial concentrations; and (V) early detection of emerging drug resistance during treatment, especially in the context of spreading less susceptible quasi-species [34,35].

In this review, we highlight the conventional and current detection approaches for the identification of antibiotic-resistant bacteria. We aim to enhance the comprehension of how rapid AST can contribute to the diagnosis of infectious diseases. We discuss the advantages and drawbacks of existing AST techniques, and we introduce key technologies that are compatible with point-of-care testing (POCT). Additionally, we speculate on the AST technologies that are expected to flourish in the future.

## 2. Traditional Culture-Based Methods

### 2.1. Conventional Antibiotic Susceptibility Testing (AST)

The European Committee on Antimicrobial Susceptibility Testing (EUCAST) and the Clinical and Laboratory Standards Institute (CLSI) have recommended the use of phenotypic testing for accurate antibiotic resistance diagnosis. This research-based approach involves determining whether a bacterium multiplies when an antibiotic is added, regardless of the resistance mechanism. These tests help determine which antibiotics are effective and what doses should be administered during therapy. Traditional AST procedures, such as disk diffusion, broth microdilution, gradient tests, breakpoint tests, and agar dilution, rely on exposing bacterial isolates to a series of antimicrobials and visually detecting growth.

The minimum inhibitory concentration (MIC) is the principal approach frequently utilized for determining antimicrobial sensitivity. This approach aims to measure the lowest antimicrobial concentration that restricts visible bacterial growth when conducted in either agar or broth. In this method, solutions containing a specified number of bacteria (typically 0.5 according to the MacFarland standard) are introduced onto agar or broth containing diluted antimicrobial concentrations [36].

Following the designated incubation period, the presence or absence of microbial growth is observed. This straightforward and cost-effective method does not necessitate specially designed equipment. The therapeutic antimicrobial concentration can be modulated for optimal therapy once the MIC has been ascertained. Nonetheless, the effectiveness of this approach is constrained when it comes to assessing resistance in non-cultivable live bacteria, and its success depends on factors such as the duration of incubation, the concentration of diluted antimicrobial agents, and initial bacterial inoculum. It is, overall, a semi-quantitative technique that may not yield a precise MIC value [36].

The disc diffusion method is a laboratory technique that involves placing a paper disc coated with an antimicrobial substance onto a solid agar medium. This leads to the formation of a circular area without microbial growth, referred to as the zone of inhibition, surrounding the disc. This zone indicates the degree of inhibition of microbial growth. This qualitative approach categorizes samples as resistant, intermediate, or susceptible. It is a practical and easily executable technique, particularly suited for rapidly growing bacteria. However, limitations include the use of antimicrobial agents that exhibit poor diffusion in agar and difficulties encountered in interpreting results for fastidious and anaerobic microbes [37].

The E-test^®^ combines elements of the previously mentioned methods, resembling disk diffusion but yielding MIC data. The experimental procedure involves the placement of a rectangular device onto an agar plate. One side of the device is designed to create a concentration gradient of antimicrobial agents, while the other side is equipped with a scale for interpretation purposes. Despite sharing the time-related limitations of the prior tests, the E-test^®^ employs an immobilized antimicrobial gradient indicated on a ruler, offering a simpler means to directly quantify microorganism susceptibility. This is particularly advantageous for challenging-to-culture organisms like *Mycobacterium bovis* and *Haemophilus influenzae*, as well as anaerobes.

Research involving the propagation of bacteria sourced from animal-based food (ABF), especially within the meat distribution system, has been widespread globally. These studies reveal the consistent presence of various microorganisms that are resistant, often to multiple antimicrobials. Notable bacteria in ABF encompass *Vibrio parahaemolyticus*, *Enterococcus faecium*, *Enterococcus faecalis*, *E. coli*, *Bacillus cereus*, *Listeria monocytogenes*, *S. aureus*, *Yersinia enterocolitica*, *Campylobacter* spp., and *Salmonella* spp. Previous investigations have highlighted resistance to numerous antimicrobial classes, such as ciprofloxacin, tetracycline, benzalkonium chloride, gentamicin, chloramphenicol, enteromycin, cadmium chloride, methicillin, streptomycin, ampicillin, sulfafurazole, nalidixic acid, vancomycin, sulfonamides, clindamycin, and amoxicillin [38].

Irrespective of the merits and demerits of conventional techniques for detecting AMR in bacteria, they play a crucial role in selecting the most optimal therapy against resistant bacteria in clinical diseases. Presently, the medical and scientific communities unanimously acknowledge that prophylactic antimicrobial use, even as growth promoters, is the primary driver of increased resistance across multiple antimicrobial classes. Prudent utilization of these medicinal products is vital, both in production and prophylaxis for animals, adhering to correct dosages and frequencies. Such caution helps prevent interference with human infection treatment and curtails the prevalence of species resistant to multiple antimicrobial drug classes. Despite reported shortcomings, traditional methods remain prevalent for identifying AMR due to their simplicity. Even studies utilizing molecular techniques initially resort to these conventional methods for their ease of use and ability to provide an overall insight into the presence of target AMR and microorganisms.

### 2.2. Advancements in Culture-Based Techniques

The use of modern optoelectronic systems, fiber optics, microfluidics, and indicator dyes that are sensitive to the redox state or pH can improve the sensitivity and overall performance of optical systems used for testing purposes [39]. Automated AST systems that have received approval from the Food and Drug Administration (FDA), including the MicroScan WalkAway 205 (Siemens Healthcare Diagnostics, West Sacramento, CA, USA), BD Phoenix Automated Microbiology System (BD Diagnostics, Becton Dickinson, San Diego, CA, USA), Vitek 2 System (bioMérieux, Craponne, France), and Sensititre ARIS 2X (Trek Diagnostic Systems, Brooklyn Heights, OH, USA), are of significant importance in clinical laboratories. These systems are equipped with diverse antimicrobial panels tailored for Gram-positive and Gram-negative pathogens. For example, these systems assess turbidity in liquid cultures automatically from multi-wells and use a redox indicator to detect microorganisms. Some systems, such as the Alfed 60 ASTTM system from Alifax in Italy, utilize highly sensitive laser-light scattering technology to identify bacterial proliferation in liquid culture broth. This device enables the rapid retrieval of antibiotic susceptibility information directly from positive blood culture bottles within a time frame of 4–6 h. These broth dilution-based devices employ pre-manufactured AST cassettes or cards that consist of positive controls and wells with escalating doses of antibiotics. They continually observe the growth’s development and examine minimal inhibitory concentration (MIC) trends for large groups of organisms using their extensive databases [40].

The Microscan WalkAway functions as a reader system coupled with an incubator, allowing for the continuous monitoring of results through photometry or fluorometry. The system has the capability to evaluate a range of 40–96 microdilution trays that consist of fixed doses of antimicrobial agents. These trays are manually inserted with bacterial cultures. The MIC results may be achieved within a very brief time frame of 7–18 h, with the duration depending on the classification of the pathogens as either Gram-negative or Gram-positive [41].

The BD Phoenix Automated Microbiology System is equipped to analyze a total of 99 test panels, each containing 84 wells with dilutions of antimicrobial agents. Growth monitoring is performed using both a turbidimeter and a calorimeter. This system can determine MIC values for various pathogens, including Gram-negative, Gram-positive, *Streptococcus pneumoniae*, β-hemolytic, and viridans, within a time frame of 6–16 h [41].

The Vitek 2 System is an advanced automated platform that utilizes miniaturized reagent cards containing both antimicrobials and testing media, which are distributed across 64 wells. It has the capacity to simultaneously conduct 30–240 tests within a span of 4–10 h, covering Gram-negative, Gram-positive, and *S. pneumoniae* pathogens [41].

The Sensititre ARIS 2X operates as an automated system based on fluorescence. It necessitates an 18–24 h incubation period to assess growth for MIC determination. Notably, the Sensititre ARIS 2X includes an autoinoculator, which serves to minimize human error. This system is suitable for assessing Gram-negative, Gram-positive, *S. pneumoniae*, *Haemophilus* species, and non-fermentative Gram-negative bacilli [41].

## 3. Molecular Techniques

Molecular-based techniques have advantages over phenotypic assays in detecting antibiotic resistance genes (ARGs), such as multiplex targeting, giving more accurate identification and detection. These techniques offer a viable alternative in certain taxonomic units where susceptibility breakpoints have not been defined. Non-purified polymicrobial samples can also be used in molecular-based approaches, which can lead to faster response times to newly added resistance factors [40]. However, molecular-based techniques have limitations, such as the inability to determine MICs and the possibility of missing some ARGs due to their limited sensitivity and coverage. Moreover, developing molecular-based assays for detecting the broad diversity of AMR genes can be costly [42]. Nonetheless, molecular-based approaches are continually being improved, using amplification and nucleic acid hybridization techniques to enhance the detection of ARGs and their expression. Overall, molecular-based techniques provide a quick and sensitive detection of ARG [43].

### 3.1. Nucleic Acid Amplification Technology (NAAT) in AST

Nucleic acid amplification testing (NAAT) is a highly robust method for detecting pathogens, particularly when used in combination with a syndromic strategy. Many diagnostic panels offered by companies like Bosch, BioMérieux, Eplex, Elitech, Becton Dickinson, and Qiagen include the detection of specific ARGs. They provide therapeutically useful data, particularly when a comprehensive antibiogram is not required. However, the detection of specific ARGs does not necessarily confirm antibiotic resistance. The identified ARGs may not exhibit a direct correlation with the pathogen responsible for the illness, or the resistance gene detected may not possess functional capabilities. NAAT does not determine MICs or provide explicit recommendations for antibiotic use. One notable benefit of NAAT is its capacity prompt updates in response to the emergence of novel pathogens and resistance factors [39]. Examples of NAATs used for antibiotic resistance detection include Polymerase Chain Reaction (PCR), multiplex PCR, Reverse Transcriptase Polymerase Chain Reaction (RT-PCR), Real-Time PCR (qPCR), and Isothermal Amplification Methods.

#### 3.1.1. Polymerase Chain Reaction (PCR) and Multiplex PCR

PCR is an in vitro technology that permits the exponential amplification of specified sequences of DNA and RNA. However, false-positive results can occur due to contamination or cross-reactivity with other related organisms or genes. To address this, various quality control measures have been developed, including the use of negative controls and the validation of assay specificity and sensitivity. They have also been used for the environmental monitoring of AMR genes in soil, water, and food samples. However, PCR-based methods have limitations in terms of their ability to detect unknown or novel resistance genes and their dependence on the availability of specific primers for target genes [44]. Unlike conventional culture methods, this approach can amplify genes from non-cultivable or dead microorganisms that might otherwise go unrecognized by standard techniques [38,45]. Despite these limitations, PCR remains a valuable tool in AMR research and surveillance. These are just a few examples, as there are many different AMR genes that can be detected using PCR: *mecA* gene, which confers resistance to methicillin in *Staphylococcus aureus* [46]; *blaSHV* gene, which confers resistance to beta-lactam antibiotics in *Enterobacteriaceae* [47]; *tetM* gene, which confers resistance to tetracycline antibiotics in many bacterial species [48]; and *vanA* gene, which confers resistance to vancomycin in *Enterococcus* species [49].

Multiplex reaction in PCR may be optimized by an upgraded technique with respect to the traditional PCR, as well as qPCR. This experimental procedure involves the use of several primers within the solution mixture, enabling the detection and differentiation of multiple microbial species during a single run. The main advantage is the decreased cost and time required with the simultaneous amplification of various genes [50]. PCR has been shown to be a good way to find point mutations in broad-spectrum AMR genes, as long as either of the primers is designed to bind at sites where the sequence changes [38,51]. Multiplex PCR techniques have also been devised to swiftly and simultaneously detect multiple pathogens in clinical specimens, along with the identification of AMR genes within the pathogens. Examples of multiplex PCR techniques for detecting AMR genes encompass the RespiFinder SMART 22 assay [52], the SuperBug ID assay [53], the Allplex™ Gastrointestinal Panel Assay [54], and the BD MAX™ MDR-TB assay [55].

#### 3.1.2. Reverse Transcriptase Polymerase Chain Reaction (RT-PCR)

The process of RT-PCR involves transcribing an RNA molecule into a complementary DNA molecule (cDNA) and amplifying it using PCR. This technique is known for its excellent specificity, sensitivity, and reliability [45,56]. Compared to DNA molecules, cDNA molecules generated from the original RNA have a higher degree of purity, as they lack contaminants such as proteins that may affect the accuracy of the test. As a result, cDNA is more specific and can be more easily detected by primers. In addition, RT-PCR can identify reproducing cells with great sensitivity, making it useful for detecting live bacteria in samples that may be contaminated with antibiotic-resistant bacteria (ABF). This is particularly important in identifying the risk of consuming ABF that can cause difficult-to-treat illnesses due to high levels of ARGs. RT-PCR is also used to qualitatively examine gene expression and is necessary for other molecular methods, such as qPCR for measuring RNA levels and microarray for identifying multiple target gene expressions [57,58]. In eukaryotic cells, RT-PCR can distinguish between exons and introns and is useful for identifying genetic disorders and evaluating antimicrobial drug therapy [58,59]. However, a significant drawback of this technique is the instability of RNA molecules, which makes sample processing challenging and requires experienced and well-trained personnel. Consequently, RT-PCR analyses can be time-consuming and expensive [60]. The specific genes targeted for detection may vary depending on the type of antimicrobial resistance being investigated and the bacterial species under study.

To assess the effectiveness of antibiotics against *Chlamydia* spp., Khan et al. [61] developed a RT-PCR assay, comparing it with standard immunofluorescence (IF) staining tests. The RT-PCR method revealed higher minimal inhibitory concentrations (MICs) of antimicrobial drugs for a *Chlamydia pneumoniae* test strain compared to IF staining, signifying its enhanced precision. Specifically, doxycycline and tetracycline exhibited the lowest MICs at 1 mg/L, while erythromycin and ciprofloxacin had higher values at 1.6 and 16 mg/L, respectively. On the other hand, Cangelosi et al. [62] pursued a new approach for assessing resistance in *M. tuberculosis*. They introduced a RT-PCR probe assay tailored to the precursor rRNA of *M. tuberculosis*. Precursor rRNA includes terminal stems that are excised during the formation of mature rRNA subunits. Disrupting RNA synthesis profoundly impacts the number of these stems in bacterial cells. The hybridization outcomes confirmed the assay’s specificity for *M. tuberculosis*, and it accurately predicted resistance to rifampin and ciprofloxacin, as anticipated.

#### 3.1.3. PCR Combined with Restriction Fragment Length Polymorphism (PCR–RFLP)

PCR-RFLP is a technique that involves using restriction enzymes to digest amplified DNA fragments that contain unique nucleotide sequences. This process can be used to verify the target sequence of genes that encode AMR through RFLP analysis [63]. However, PCR-RFLP is a complex and multi-step procedure that is often considered unfavorable due to the extended duration necessary for obtaining outcomes and the increased expenses associated with diverse restriction enzymes [38]. For example, the *blaCTX-M* gene, renowned for its capacity to bestow resistance against extended-spectrum beta-lactam antibiotics, was detected using PCR-RFLP. PCR–RFLP has been utilized for the identification of genetic variations in the *gyrA* gene that are related to AMR in *Campylobacter* [64]. PCR-RFLP analysis unveiled the presence of a point mutation at the Thr-86 position of the *gyrA* gene in isolates that had previously been identified as ciprofloxacin-resistant using the MIC method. Another example in *Salmonella* highlighted the effectiveness of the PCR–RFLP technique in identifying mutations within the quinolone resistance-determining region of the *gyrA* gene in nalidixic acid-resistant strains found in poultry samples, thus underscoring the potential contribution of such point mutations to AMR [65].

The mismatched PCR-RFLP assay was developed to identify mutations in *A. baumannii* associated with fluoroquinolone (FQ) resistance. Two sets of primers were designed for the detection of *gyrA* and *parC* mutations, which was achieved by including artificial restriction enzyme cleavage sites in PCR products. The assay was evaluated using a total of 58 strains of *A. baumannii* and 37 strains of other *Acinetobacter* species. FQ-susceptible strains had their PCR products digested, while FQ-resistant strains with mutations in *gyrA* and *parC* did not. Importantly, the assay could distinguish *A. baumannii* from other Acinetobacter species, making it a valuable tool for quick FQ resistance assessment in *A. baumannii* without precise species identification. However, it cannot detect mutations at other locations or in other genes, and further research is needed to confirm its differentiation capabilities. Nonetheless, it offers a rapid, specific, and cost-effective method for detecting significant FQ resistance mutations in *A. baumannii*, aiding clinical assessments and epidemiological studies [66].

#### 3.1.4. Real-Time Polymerase Chain Reaction (qPCR)

Quantitative PCR (qPCR) is a technique that can provide an estimate of the number of microorganisms present in a sample. Quantitative reverse transcription PCR (qRT-PCR) can also be employed to assess the expression level of resistance genes following exposure to varying antibiotic doses, providing an estimate of MIC values. However, the expense of qRT-PCR equipment and reagents generally exceeds what is reasonable for routine AST testing. In qPCR, the amplified fraction of DNA can be identified and measured throughout the amplification process by using fluorescent probes that release detectable signals [45,63,67]. The utilization of fluorescent probes, such as molecular beacons, in PCR amplifications facilitates real-time monitoring of the amplification process, resulting in quicker reaction times and the ability to quantify both the relative and absolute quantities of microorganisms in the sample [63]. However, qPCR equipment and reagents are more expensive than those used in traditional PCR. In addition, environmental or food matrix microorganisms that are not the focus of the research may inhibit the reaction [63]. To assess the clinical consequence of AMR, the use of RT-qPCR to measure the expression level of resistance genes is critical [38]. *blaCTX-M* gene is responsible for resistance to extended-spectrum beta-lactam antibiotics. It is frequently found in Gram-negative bacteria, such as *E. coli*, and qPCR was proven to successfully detect the existence of this gene [68]. The primers and probes used in qPCR are designed to target specific regions of the gene, allowing for its detection and quantification. Also, *mecA* gene MRSA can be detected using qPCR [69]. In addition, *tet* genes which encode resistance to tetracycline antibiotics are commonly found in various bacterial species. qPCR can be used to detect different *tet* genes, such as *tetA*, *tetB*, *tetM*, etc. [70]. Moreover, *van* genes are present in vancomycin-resistant *Enterococcus* (VRE) strains. qPCR can be used to detect *vanA*, *vanB*, and other *van* gene variants [71].

#### 3.1.5. Isothermal Amplification Methods

Recent advances in molecular biology include the use of isothermal DNA amplification removing the requirement for thermocycling, which is required for standard PCR procedures. Numerous techniques for isothermal nucleic acid amplification have been devised, including nucleic acid sequence-based amplification (NASBA), strand displacement amplification (SDA), rolling circle amplification (RCA), transcription mediated amplification (TMA), recombinase polymerase amplification (RPA), helicase-dependent amplification (HDA), and loop-mediated isothermal amplification (LAMP) [72]. These methods paved the way for the development of cutting-edge, next-generation molecular diagnostics [73]. Yamamoto et al. [74] proposed a technique utilizing LAMP for the detection of carbapenem-resistant *A. baumannii* (CRAb) strains carrying the *blaOXA-23* gene.

Furthermore, Poirier et al. [75] introduced the creation of LAMP rapid diagnostic assays. These assays were devised for the swift detection of both *Klebsiella pneumoniae* and carbapenemase genes within clinical samples [75]. A significant advantage of isothermal methods, as opposed to conventional PCR-based techniques, is the elimination of the need for thermocycling. Consequently, this leads to decreased power consumption and shorter analysis times. The reliance on thermocyclers has become outdated, given the availability of alternative temperature control methods like water baths or hotplates [76]. Furthermore, it is important to highlight that isothermal amplification, as opposed to PCR, offers improved speed and sensitivity [77]. This is because isothermal amplification does not rely on distinct heat cycles, thus enabling continuous amplification. As a result, this method can produce detectable amplicons in less than 10 min. Several isothermal approaches, such as RCA, LAMP, and HDA, provide the advantage of not requiring template denaturation and being capable of withstanding biological components, particularly in the case of LAMP and HDA [78]. While it is true that isothermal technologies such as LAMP demand more sophisticated primer design techniques, they offer improved specificity in comparison to PCR. A study on multiple isothermal approaches in terms of simplicity, affordability, sensitivity, and repeatability indicated that both LAMP and RPA methods show significant promise for point-of-need (PON) diagnostics, particularly in low-resource settings. Both methods employ a single-step process, involving incubation at a specific temperature, and they require minimal amounts of DNA template [76]. Furthermore, isothermal methods offer advantages for microfluidic-based techniques for the same reasons mentioned above [79]. It is worth noting that LAMP amplicons have the ability to be visually detected without the use of any specialized equipment, as evidenced by the observation of turbidity or color change [60]. However, isothermal techniques also possess significant limitations. The efficiency of multiplexing approaches in isothermal procedures is diminished because of the heightened complexity of the experimental design [80]. Furthermore, it is important to acknowledge that numerous isothermal amplification techniques involve intricate reaction processes, often demanding a significant number of primers. For example, the LAMP approach requires the use of 4–6 primers, whereas other techniques, like NASBA, entail multiple enzymatic phases [28,78]. Nevertheless, in addition to the speedy and simultaneous provision of trustworthy data, isothermal PCR has been observed to exhibit numerous advantageous characteristics, including cost-effectiveness, high sensitivity, specificity, ease of use, minimal spatial requirements, and widespread accessibility [35,81,82,83,84].

### 3.2. Next-Generation Sequencing (NGS)

NGS enables the rapid analysis of bacterial genomes within hours. A diverse range of technology solutions have been launched, including benchtop equipment with dimensions similar to a laser printer, such as 454 GS Junior (Roche, Basel, Switzerland), MiSeq (Illumina, San Diego, CA, USA), and Ion Torrent PGM (Life Technologies, Grand Island, NY, USA). In prior research, the MiSeq (Illumina) has exhibited a superior performance, boasting higher productivity per run and lower error rates. The results interpretation of whole bacterial genomes is typically carried out through either allelic comparisons [85] or the examination of single nucleotide polymorphisms (SNPs) [86]. Commercial software programs such as SeqSphere+ version 6.0.2 or BioNumerics version 8.1.1 have the potential to enhance the process of data assessment and interpretation.

The NGS permits resistance detection based on the existence of the underlying mechanism, as opposed to pharmacodynamic criteria alone [87], and, as such, may eventually transform microbial resistance testing. NGS has gained substantial recognition in the realm of resistance testing, particularly for infectious agents with slow growth and unconventional resistance patterns, such as *Mycobacterium tuberculosis* strains resistant to multiple drugs or extensively resistant (XDR). The capability to swiftly detect or exclude resistance determinants through NGS has profoundly influenced the therapeutic approaches adopted for these strains. The comprehensive genetic analysis within five days enabled accurate resistance discovery in the complete genome of *M. tuberculosis* isolates from Burmese, Hmong, and Indian immigrants living in the USA [88]. Similar genome sequencing results were presented for drug-resistant strains from Russia, which contained nearly all known mutations related to drug resistance [89]. However, resistance testing based on NGS is not limited to mycobacteria. NGS was utilized to detect transmissible plasmids in multidrug-resistant *E. coli* isolates with an ESBL phenotype. These isolates were consistently observed to transfer their cefotaxime resistance marker during laboratory conjugation studies [90]. In the course of epidemics, high-throughput sequencing proves to be a helpful tool for tracking resistant plasmids [91]. Nonetheless, while the commercial NGS assay known as the Hospital Acquired Infection BioDetection System, developed by Pathogenica in Boston, MA, USA, has proven effective in detecting outbreaks caused by Enterobacteriaceae strains carrying Extended-Spectrum Beta-Lactamase (ESBL), it has been observed to lack the capability to distinguish between ESBL and non-ESBL TEM and SHV β-lactamases or to identify specific CTX-M gene groups [92]. The costs and paucity of user-friendly bioinformatics platforms are now impediments to the widespread application of the NGS technology in microbiological diagnostic and resistance screening [93]. NGS methods deliver high-resolution genotyping in a short time frame of about two to five days [93]. Hence, NGS is poised to be the logical evolution for routine microbiological laboratory diagnostics of bacterial diseases and the forecasting of antibiotic susceptibility [94], probably replacing traditional cultural practices in the medium-to-long term [35]. In addition, NGS is able to characterize new resistance mechanisms when they are found. This may be accomplished by sequencing isolates that have already been shown to be phenotypically resistant, offering a significant advantage over other nucleic acid-based approaches [28].

#### 3.2.1. Whole-Genome Sequencing (WGS)

In light of the current advancements in the cost-effectiveness of sequencing technology, WGS has become a readily available and useful tool for combating antibiotic resistance, a significant challenge to modern healthcare. WGS has made substantial progress in this field, ranging from the creation of innovative antimicrobial drugs and diagnostic tests to real-time surveillance. It has also contributed to our understanding of the factors that enable the emergence and persistence of resistance [38].

WGS-AST has evolved as a rapid and precise approach for detecting AMR. In certain instances, there is a clear correlation between genotype and phenotype, allowing the detection of specific AMR genes using clinically applicable molecular testing. However, a majority of antimicrobial resistance mechanisms entail multiple genes and intricate cellular signaling pathways, and many of them are not fully comprehended. Hence, the field of WGS offers an alternative approach for grasping these mechanisms. By capturing the entire genome of pathogens isolated in clinical settings, WGS enables a thorough identification of AMR. Furthermore, it has been reported by Yusof et al. [28] that WGS is one of the predominant approaches employed across the studies included in the systematic review and meta-analysis for identifying mutations in colistin resistance genes among *K. pneumoniae* isolates was WGS.

The WGS of DNA taken from the tested samples is assembled using programs such as SPAdes, Velvet, ABySS, and SOAPdenovo, which are based on a De Bruijn graph. Small sequencing reads may be assembled into contigs, which can be annotated to seek resistance genes. WGS does have one major drawback, however: it generates vast quantities of data, making it impossible to forecast the presence of undiscovered AMR genes or gene variations associated with antibiotic resistance. Fortunately, the datasets derived from the application of WGS to clinical isolates can be effectively analyzed using various freely available bioinformatic tools. These tools include ResFinder, AMRfinder, Comprehensive Antibiotic Resistance Database (CARD), ARG-ANNOT, ARGs-OAP, RGI, ARGs-OAP (v2), ARIBA, PointFinder, NCBI-AMRFinder, SRST2, SEAR, ShortBRED, PATRIC, SSTAR, KmerResistance, GROOT, and DeepArgs, among others. These tools are actively curated and offer the capability to identify genetic elements associated with antimicrobial resistance (such as SNP mutations, horizontal gene transfer, inversions, etc.) without necessitating extensive expertise in bioinformatics. For example, a draft genome of *E. coli* INF191/17/A was predicted as an extended-spectrum beta-lactamase (ESBL) strain carrying fourteen antibiotic resistance genes, using the ResFinder database [95]. The choice of database is dependent on the goal of each study (i.e., resistance genes, virulence genes, and proteins), as well as the sequence confidence contained in each database [96].

WGS used prediction tools that were dependent on large, high-quality AMR gene databases to determine and forecast the existence of AMR genes and other AMR mechanisms in a particular clinical isolate. The disparities among these tools are predicated upon the algorithms employed by their respective databases and the composition of their data. While numerous studies have highlighted the effectiveness of bioinformatic resources in detecting AMR, a recent inter-laboratory study conducted by Doyle and O’Sullivan [97] unveiled notable disparities among the results among various laboratories. These discrepancies were attributed to the varied software and analytical pipelines utilized for the identification and prediction of AMR gene presence or absence in identical clinical isolates. Consequently, a lack of alignment with phenotypic AST results was observed, signifying suboptimal concordance. The inconsistent findings seen in this study can be attributed to several factors, including the choice of bioinformatics tool, the quality of the WGS sequences, and the interpretation of the data obtained from the bioinformatic analysis.

The primary advantage of WGS-based techniques for studying AMR is that they may concurrently examine all the components involved in the establishment of the phenotypic and determine the link between each ARG and mobile or nonmobile elements harboring it. WGS also paves the way for genome-wide studies to be developed from the examination of several genes and mutations [85,86,98,99,100,101,102,103]. Katiyar et al. [104] employed WGS to conduct a thorough investigation of AMR genes, aiming to identify potential associations with phenotypes that could enhance the selection of more precise treatments. Notably, their research revealed that all fluoroquinolone-resistant strains exhibited mutations in the *gyrA*, *gyrB*, *parC*, and *parE* genes. This study advances our understanding of WGS’s capacity to predict resistance genotypes and their links to phenotypic traits, enabling the rapid detection of AMRdeterminants and facilitating targeted antibiotic usage directly guided by genetic sequences [105]. Moreover, WGS is crucial for conducting phylogenomic investigations encompassing the accessory genome that goes beyond the standard multi-locus sequence typing (MLST) analysis, which takes into consideration simply the core genome. The acquisition of virulence determinants and AMR is a crucial factor in the evolution of bacterial development [106,107]. Therefore, the utilization of WGS techniques is of utmost importance in comprehending the fundamental elements involved in the progression of virulence and AMR. In addition to their use in epidemiology, WGS methods play a crucial role in elucidating emerging mechanisms of resistance and predicting the potential evolution of resistance, including resistance to therapies that are not yet accessible on the market. Here, it needs to be highlighted that epidemiological investigations must inevitably rely on what is known. The discovery of new resistance mechanisms is typically laborious, and WGS techniques help tackle this challenge. Indeed, the use of these technologies is progressively expanding in the identification of genes associated with AMR, facilitating the creation of genome repositories and the annotation of genes derived from strains that are clinically significant [108].

In the United States, the national laboratory capabilities for monitoring AMR and conducting WGS are on the rise and expanding, consisting of networks run by universities and state public health laboratories under federal management. The Centers for Disease Control and Prevention (CDC) oversees the Antibiotic Resistance Laboratory Network (ARLN) to quickly identify the developing resistance risks in relation to healthcare settings, food, and the general public. This vast network executes WGS for a wide range of pathogens, encompassing all isolates of *M. tuberculosis*; *Neisseria gonorrhoeae*; and other important pathogens, such as those linked to outbreaks. One further limitation of WGS methods is their typical reliance on the isolation of the organism. In order to overcome this challenge, metagenomic analyses such as Nanopore, which is portable, rapid, and produces long reads, can be utilized, particularly in microbiomes with low complexity.

#### 3.2.2. Metagenomics for Antimicrobial Surveillance

The main focus of metagenomic approaches is short-read NGS data, which make it possible to measure hundreds of transmissible resistance genes in a single sample without having to use any predefined genes. Consequently, these data will provide more insight into the types of bacteria, diseases, and virulence genes that are present, and the collected data may be reanalyzed in the future if new genes of interest are discovered [109]. It has been shown that metagenomics is superior to conventional methods of AMR surveillance in pig herds [110], and it has proven to be an efficient tool for comparing AMR in animals [111], as well as for investigating epidemiological data [112]. Due to its various benefits, metagenomics is widely regarded as a promising method for AMR surveillance. Eventually, it is plausible that a centralized surveillance system for AMR may emerge that facilitates the detection of all resistance genes [8,109].

In *Neisseria gonorrhoeae*, an analysis of transcriptomes revealed that the transcriptional response to azithromycin is influenced by genetic distance and the population structure. When exposed to drugs, transcripts for rpsO, rplN, omp3, and NGO1079 exhibited the most significant changes between phenotypes [113].

The expression of almost half of the genes involved in multidrug resistance in *Salmonella enterica serovar* Typhimurium was altered after exposure to subinhibitory doses of chlortetracycline and florfenicol. Notably, there was an increase in genes associated with adhesion function and those located inside *Salmonella* pathogenicity islands subsequent to drug exposure [114].

Metagenomics has emerged as a valuable tool for the identification and analysis of pathogens and their genotypic resistance [56], and it has the potential to delineate microbial communities without the need for traditional culture-based methods [115,116]. This approach involves the sequencing of the entire genetic material of the community. Boolchandani et al. [96] conducted a comprehensive analysis to elucidate the inherent benefits and drawbacks associated with AMR gene databases. Metagenomic sequencing has been found to be highly effective in the analysis of microorganism ecology [67,117]. Along with NGS, a comprehensive database of sequences from diverse contexts is acquired [118]. Although it has the disadvantage of being a pricey technology, it is a solid choice for providing the capability for an extensive analysis in research involving bacterial resistomes [119]. Furthermore, metagenomics, being an open-approach molecular technique, is subject to some constraints. One such drawback is the potential inadequacy in providing comprehensive sequencing of a species’ genome, particularly when the samples originate from intricate communities like those seen in soil ecosystems [38].

### 3.3. DNA Microarray

Microarray technology is a noteworthy avenue to be acknowledged in the context of analyzing genetic AMR in bacteria. The utilization of this methodology enables the examination of gene expression through the process of hybridizing oligonucleotide sequences that serves to purify and amplify specific RNA molecules from the targeted sample. This permits its usage for numerous applications, especially for determining the function of specific genes [120,121]. This method has been widely recognized as the most effective method for studying transcriptomes. The application of microarray technology for the identification of AMR genes can be maximized by various hybridization techniques, allowing for the simultaneous analysis on a single substrate (such as glass, membrane, or gel pad) containing several probes [121,122,123]. Another important benefit is that preceding cultivation of bacteria is not essential, as the DNA sample may be immediately isolated to generate the microarrays [124]. Microarray usage has been superseded in recent years by NGS approaches. One disadvantage of microarrays, for example, is the necessity for the previous information on the genetic areas to be investigated. In addition, by analyzing the selected target locations alone, one may end up missing vital and critical information in the samples. Another key challenge is the hybridization of identical sequences, hindering the reading and analysis of target genes. An example of a DNA microarray is the one developed by Frye et. al. [125] to detect ARGs identified in the National Center for Biotechnology Information Database.

### 3.4. Fluorescence In Situ Hybridization (FISH)

The conventional method of FISH relies on the targeted binding of fluorescently labeled, single-stranded oligonucleotide probes, typically ranging from 18 to 25 bases in length, to the ribosomal RNA (rRNA) of the desired organism. This technique is then followed by an analysis using a fluorescence microscope, which enables the identification of microorganisms at either the genus or species level. In theory, it is possible to hybridize FISH probes with many types of intracellular RNAs. However, rRNA is particularly advantageous as a target for FISH due to the abundance of ribosomes in a cell involved in protein synthesis, which enables the amplification of fluorescence intensity [126]. This classic FISH approach is both speedy and easy to standardize and may be employed as such for molecular rapid testing. Minor alterations to the procedure entail the use of commercially patented peptide nucleic acid (PNA) probes or probes that integrate locked nucleic acids (LNAs) as opposed to conventional single-stranded DNA probes. The PNA-FISH approach is advantageous in reducing nonspecific probe attachment mainly because of the electrically neutral backbone of the oligonucleotides. This technology is particularly suitable for routine diagnostic purposes due to its improved level of consistency. However, PNA probes that are covered by patents tend to have a high cost [127]. FISH is highly suited for the discovery of resistance determinants if two conditions are met. Ribosomally mediated resistance, including its influence on antibiotic drugs such as macrolide or linezolid, is particularly advantageous due to the abundance of rRNA copies in viable cells, thus facilitating the generation of distinct fluorescence signals. Moreover, FISH may be utilized efficiently in cases when just a limited number of variable bases exhibit resistance. This implies that a probe panel with a high number of probes is not necessary. The initial study of resistance testing based on FISH for mechanisms of resistance unrelated to those relying on rRNA has been reported [128]. FISH technology has advanced to include signal-amplified, catalyzed reported deposition (CARD) FISH; doubly labeled oligonucleotide probe-based (DOPE-based) FISH; combinatorial labeling and spectral imaging (CLASI) FISH; and the combination of FISH with other diagnostic approaches, as well as FISH procedures for gene identification which require in situ amplification of the respective gene, as in case of the RCA FISH [129]. Some examples of AMR genes that can be detected by FISH include *blaCTX-M* [130], *mecA* [131], and *vanA* [132].

The rapidity, simplicity, and low cost of the FISH-based identification of resistance determinants make it a potentially useful diagnostic method. The concomitant quick diagnosis of antimicrobial resistance can provide a prompt adjustment of antimicrobial therapy, resulting in potential benefits for the patient’s well-being. The key benefit of FISH is its potential application for resistance testing directly from primary materials, including tissues, with minimum effort. Hence, FISH may also be utilized in resource-limited environments where pricey technologies are not accessible. In contrast to PCR, FISH may also ascribe a particular resistance mechanism to bacteria. However, thus far, FISH is confined to only a few indications for which methods have been published [35]. As an additional issue, standardization of FISH-based resistance testing is often absent. If used from primary sample materials like tissue, tissue autofluorescence has to be addressed, requiring some knowledge to understand such diagnostic results. To minimize the potential for misinterpretations, it is essential to conduct counterstaining with a pan-eubacterial FISH probe and nonspecific DNA staining when conducting a FISH analysis on tissue samples. These steps are crucial for confirming the presence of nucleic acids associated with the identified infections [133]. Considering the aforementioned constraints, it is likely that FISH for resistance testing will serve as an interim method until amplification-based solutions are readily available as user-friendly and economically viable benchtop systems in the commercial sector [35].

## 4. Innovative Approaches

### 4.1. Mass Spectrometry-Based Methods

Matrix-assisted laser desorption/ionization–time-of-flight mass spectrometry-based (MALDI-TOF MS-based) intact cell mass spectrometry (ICMS) has become the standard approach for identifying species of cultured bacteria and fungi [30,134,135,136,137,138]. Using ICMS spectra, promising methods have been developed for subspecies identification [139]. In contrast to what is now available in clinical routine diagnostics, this method shows great potential for the rapid detection of susceptibility-linked biomarker ions. The ability to demonstrate phylogenetic relatedness using phyloproteomic methods allows for the indirect discovery of predominantly chromosomally encoded resistance genes [140,141,142,143,144,145]. Alterations in the bacterial or fungal proteome caused by exposure to antimicrobials can be detected by MS [30,146,147,148]. Stable isotope-labeled amino acids (SILACs) provide an additional method for detecting global proteome modifications resulting from antimicrobial exposure [149,150].

Antibiotic resistance may now be detected using MALDI-TOF MS, which is a rapid and reliable method for identifying microorganisms in clinical isolates and clinical samples. There are three methods for using MALDI-TOF MS to detect AMR: (1) detecting AMR by analyzing at pathogen peak patterns, (2) assessing antibiotic changes owing to enzyme activity, and (3) quantifying bacterial growth while exposed to an antibiotic [151]. Numerous studies have showcased MALDI-TOF MS’s ability to identify AMR in clinical isolates. However, this method is not extensively adopted, due to its limited sensitivity in directly detecting AMR from clinical samples and its capability to test only a small number of AMR mechanisms. However, in recent years, machine learning methods have been used to identify drug-resistant bacteria, using MALDI-TOF MS profile spectra [145].

The MALDI-TOF MS technique is highly regarded for its reliability, rapidity (delivering results within minutes), accuracy, ease of use, cost-effectiveness, and environmentally sustainable attributes [152]. Despite the significant reduction in turnaround time (TAT) for bacterial identification and advancements in the determination of AMR, the implementation of MALDI-TOF MS systems in low-resource settings or as a point-of-care (POC) platform for AMR or AST is hindered by the substantial cost and large size of these systems [153]. In addition, MALDI-TOF MS is not ideal for the characterization of mixed materials due to the necessity of purification, culture, and sample preparation. In addition, other chemicals, such as the matrix, are needed to conduct the tests [154]. Available databases should provide spectra that can distinguish between susceptible and resistant strains [26]. Some examples of AMR genes that can be detected by MS include *mecA* (methicillin resistance in *S. aureus*), *blaKPC* (carbapenem resistance in *Enterobacteriaceae*), and *vanA* (vancomycin resistance in *Enterococcus* species) [155,156,157].

### 4.2. Bioinformatics Approach for Detection of AMR Genes and Databases

The field of bioinformatics encompasses a range of computer methodologies, such as sequence and structural alignment, as well as the analysis of extensive biological datasets, including genetic sequences, cell populations, and protein samples. These approaches are employed to create novel predictions or uncover previously unknown biological phenomena [158]. As more and more data become available in molecular biology, genomics, transcriptomics, and proteomics, the utilization of bioinformatics tools and techniques to comprehend this information is gathering momentum [109]. The explosion in the quantity of data stored in databases and the published literature has been used in creating molecular profiles and studying the epidemiology of infections. Consequently, it is crucial to use bioinformatics tools and techniques for controlling bacterial resistance, identifying and classifying pathogens, locating markers for early diagnosis and treatment, facilitating personalized therapies, and predicting patient outcomes [159].

Bioinformatic techniques may allow healthcare systems globally to have stronger control over AMR. To this end, techniques such as MALDI-TOF-MS coupled with WGS, which are progressively becoming part of the standard procedures in many microbiology laboratories, can assist us not only in the detection but also in gaining a deeper understanding of the diverse mechanisms of AMR in bacteria and other pathogens. Moreover, with the assistance of bioinformatics, we can evaluate the large quantity of information collected in order to select particular methods, adapted to a given situation, to enhance preventive, monitoring, and therapy measures for diseases due to antibiotic-resistant bacteria [160].

Bioinformatics methods are employed to investigate antibiotic resistance, utilizing computer algorithms capable of predicting, identifying, inferring, and assessing antibiotic resistance genes (ARGs) based on data obtained from isolated cultures. These algorithms are applied in conjunction with systematic aggregations of existing knowledge gathered from databases.

In contrast to current prediction methodologies, novel detection techniques employ rule-based inference, leveraging pre-existing knowledge about AMR phenotypic characteristics. There are also hybrid methods that combine knowledge-based detection with machine learning and mathematical inference [161]. Specifically, Resfams addresses the challenge of distant homology detection by utilizing hidden Markov models for the identification of AMR proteins [162]. Despite the effectiveness of these methodologies, it is crucial to remember that forecasts require validations. Existing approaches for predicting AMR vary in terms of their connection to prior knowledge sources (such as database-based methods), availability (standalone or online service), and the scope of the data that they utilize (ranging from gene or protein sequences to entire genome or proteome data) [162]. Some prediction approaches based on databases rely on genome assembly, while other programs permit raw sequences or protein sequences as input data. Contig generation is required for analyzing the structure of genetic elements containing ARGs, while the utilization of raw sequences may be more suitable for measurement purposes [163].

A fundamental limitation to the widespread adoption of these techniques as diagnostic tools is that they only give predictions. The relationship between genotype and phenotype is significant for mutation-based resistance [163], and informatics has made progress in linking genotypic and phenotypic data [164]. In the case of resistance genes, it is essential to observe that the manifestation of resistance is influenced not only by the specific ARGs but also by its genomic context. In addition, sequence-based methods cannot detect previously unknown resistance mechanisms that might play a role in the phenotype [165,166]. In addition to in-house Blast search approaches, tools linked to specific databases such as ARIBA [167], ARG-ANNOT [168], AMRFinder [169], MEGARes [170], CARD [171], or Resfinder [172] have been productively utilized to discover potential resistance genes and AMR mutations in a range of genomes and metagenomes [173].

The different bioinformatics applications have the capability to process sequence data in two distinct formats: reads or assemblies [96]. When employing assembly-based procedures, discrepancies across assemblers may undermine the comparability of the output [174,175]. After the assembly process is completed, BLAST and hidden Markov model searches, among other methods, are often used to compare the input data with the AMR reference databases. The results generated by BLAST-based programs can vary depending on the default settings, such as the gene size and percentage of identity. This might negatively decrease specificity if the parameters are too low or too high. Moreover, assembly-based approaches need extensive computer resources. Notwithstanding these constraints, assembly-based approaches may offer additional benefits in an AMR monitoring setting by permitting the analysis of the genomic background of the AMR, such as the existence of mobilizable resistance genes. Some examples of AMR genes detected by assembly-based bioinformatic approaches include *blaCTX-M*, *blaNDM*, *mcr-1*, and *tet(M)* [38]. Read-based approaches may employ multiple tools to align reads to AMR databases, including Bowtie2, BWA, and KMA [96]. The KMA (kmer alignment) has been developed to map raw reads directly against redundant AMR databases [176]. The KMA tool was built exclusively for the fast and precise analysis of bacterial genomes. This is different from other mapping approaches, like BWA, which were made for huge reference genomes, such as the human genome, and afterwards adapted experimentally to microbiology [176]. The KMA algorithm employs k-mer seeding as a means to enhance mapping efficiency, while also utilizing the Needleman–Wunsch technique to accurately align extensions derived from the k-mer seeds. The process of addressing multi-mapping readings involves the use of a distinct sorting strategy known as the ConClave scheme, which is employed to guarantee the precise selection of templates [176]. The application of read-based methodologies facilitates the detection of AMR genes, even when they occur in minimal abundance, that could be neglected if the genome assemblies are incomplete [96,109]. Some examples of AMR genes detected by read-based bioinformatic approaches include *blaSHV*, *mecA*, *tetM*, and *sulI* [38].

The efficacy of in silico AMR prediction is vitally dependent on the provision of correct datasets regarding AMR, irrespective of the bioinformatics technique utilized. The AMR reference databases are categorized into several solutions designed specifically for the identification of resistance to various antimicrobials or particular bacterial species, as well as a medium allowing for the identification of almost any potential AMR determinant in any DNA or amino acid sequence. AMR reference databases have significant variances that users must be aware of in order to be able to select the most appropriate database for their needs. First, AMR reference databases have varying standards for entry inclusion. For instance, CARD entries must be published in scholarly journals. Publication, however, is not required for entry inclusion in ResFinder. The genes need to be indexed in GenBank and vetted by a professional expert. In addition, the sorts of entries vary amongst databases, with the majority of databases including AMR genes and only a minority of them containing mutations of chromosomal genes triggering AMR. Various AMR databases exhibit distinct entry formats (such as FASTA, JSON, etc.) and diverse download alternatives and adhere to varying curation schedules [109].

Importantly, the existing available methods are capable of detecting novel gene variations, but they cannot discover new AMR genes. With the application of iterative k-mer-based analytics and other sophisticated algorithms, researchers are attempting to identify new resistance components from genomic data. However, these modalities need a well-characterized reference genome with phenotypic data for studies [177,178,179,180,181]. Since the primary purpose for setting criteria to evaluate the capacity of certain bioinformatics techniques is to deliver an accurate analysis of the AMR gene content, it is crucial that the agreement among the cutoff values and the anticipated conclusion is high [182]. Detecting quiet resistance genes in phenotypically susceptible microbes is less significant than misidentification of a resistant strain. As discussed earlier, differences between phenotypic reference results and projected genetic outcomes are frequently the result of inaccurate phenotypic AST test data. Often, the function of bioinformatics techniques is evaluated by comparing the genotypic and phenotypic results and calculating the validity, reliability, and performance before comparing these parameters across bioinformatics tools [175]. As is performed internationally with the databases for genomic sequence, synchronized harmonization of the databases is required to eliminate inconsistencies caused by algorithmic variances [109].

The Antibiotic Resistance Ontology (ARO) was established by a biocurator team from the CARD to interact with resistome analysis through the application of software development and the initiatives for prediction in the CARD’s Resistance Gene Identifier (RGI) software version 5 [171]. Significant improvements to CARD’s usability were made in 2017 with the addition of new classification paradigms and analysis tools, as well as the modification of the ontological framework and the curation of over 500 more AMR detection models [171]. Most notable is the readily accessible new module (Resistomes and Variants) that offers a statistical overview of in silico predicted resistance variants across a spectrum of 82 pathogens and encompasses a database over 10,000 genomes [171]. Incorporating these resistance variations into CARD has potential to consolidate and summarize anticipated resistance patterns, using the comprehensive data within CARD. This integration, in turn, will enable the discernment of emerging trends in AMR mobility and the identification of previously uncharacterized and new resistance variants [171]. There is a wide selection of resistance gene prediction tools, such as Resfams [183] and ARG-ANNOT [168], which are comparable to RGI in a sense that they all enable a certain level of identification of specific components of resistance [168]. Bioinformatics methods are often necessary when studying genomic sequences that can accommodate missing data, such as unsampled sequences, whose simple sequence similarity requires comprehensive evidence to predict the antibiogram [181]. The development of Probabilistic Graphical Models (PGMs), a mathematical framework for integrating uncertainty and probability when making predictions from limited or noisy data [184], has made it one of the very effective approaches in genomics research, especially in the investigation of regulatory mechanisms and the genetic architecture of diseases [159].

### 4.3. Microfluidics, Biosensors and Nanotechnology

Recent advances in microfluidics, biosensor technologies, immunodetection, and isothermal amplification-based NAAT have yielded a number of effective systems with the potential to alter the paradigms of AST. Biosensor systems that detect variation in the metabolism of microbials, movement, or heat generation demonstrate their clinical effectiveness. Fast, dependable, user-friendly, and affordable solutions that are suitable to AST in outpatient clinics remain elusive [185]. The biosensor-based AST (b-AST) system from Genefluidics Inc. (Duarte, CA, USA) measures 16S rRNA molecules to evaluate bacterial growth in Hybridization-Based Systems, using an electrochemical biosensor. This device merges nanotechnology, a plastic micro-electromechanical system, and microfluidics with species-specific probes [186].

The application of biosensor and chemosensor technologies is an additional option method that acquired a simplistic technical approach, affordable, time efficient, and easy to operate, and holds remarkable capacity for providing vital data for the collection of data in real time. This also facilitate the concurrent assessment of several analytes among the same device [187,188,189,190,191,192,193,194,195]. In contrast, chemosensors are commonly regarded as instruments utilized for the detection and quantification of signal generated by a chemical reaction. On the other hand, biosensors are described as “compact analytical tools that encompass a biological or biologically derived sensing component within or closely associated with a physicochemical transducer” [196].

Numerous instances of sensors and biosensors designed for AST and the identification of AMR genes have been extensively discussed in the scientific literature [174,175,176,177,178,179]. Notably, Bonini et al. [169] extended the frontiers of biosensing by introducing the CRISPR/Cas system as a potent methodological breakthrough in the detection of nucleic acids. In a different avenue, Koydemir et al. [197] employed Micro-Electro-Mechanical Systems (MEMS) biosensors to successfully detect MRSA. In a similar vein, Xu et al. [198] introduced an ingenious electrochemical biosensor with remarkable sensitivity for pinpointing the *mecA* gene in MRSA strains. Departing from these molecular approaches, Bhardwaj et al. [199] embarked on immobilizing bacteriophages on graphene electrodes to devise a method for the impedimetric sensing of *Staphylococcus arlettae* bacteria. Meanwhile, Gupta et al. [200] delved into a comprehensive exploration of cell-based biosensors, encapsulating current trends, hurdles, and future prospects. Lastly, in a fascinating amalgamation of techniques, Hu et al. [201] unveiled an inventive strategy for whole-cell biosensing, utilizing siderophore-based molecular recognition coupled with localized surface plasmon resonance (SPR) technology. Collectively, these examples underscore the diverse and innovative methodologies that researchers have employed to tackle the challenges of AST and the identification of AMR genes. 

Phenotypic or genotypic techniques can be used to detect AMR by using sensor and biosensor technology. While genotypic studies help discover the genes that express AMR pathways, phenotypic procedures focus on identifying how the bacterium’s resistance mechanisms manifest. Databases like CARD, ResFinder, and Gene contain a large number of identified and listed resistance target genes. Chemical biosensor development for phenotypic AMR detection has greatly increased over the past ten years [202,203,204,205].

Sensors and biosensors for AMR testing have been divided into mass, magnetic, mechanical, optical, thermal, and electrochemical categories based on the transducer used. Mechanical biosensors are tools that can translate surface-level interactions and processes into observable mechanical qualities and are sensitive to physical changes in mechanical features [202]. The measurements of reflectance, chemiluminescence, absorbance, fluorescence, Raman scattering, and surface plasmon resonance (SPR) are used by optical sensors in contrast [206,207,208,209]. SPR is among the most commonly employed optical label-free platforms [210,211]. Electrochemical biosensors can monitor potentiometric, voltametric amperometric, or impedimetric signals [188,196,203,212,213,214,215].

#### 4.3.1. AST Magnetic, Mass, and Mechanical Biosensors

In order to monitor AMR, researchers have investigated the magnetic characteristics of several materials. Asynchronous magnetic bead rotation (AMBR) has emerged as a technology for this purpose. This technique involves watching the speed at which magnetic beads rotate within a magnetic field that is constantly changing. The process of measuring focuses on the differences in rotation frequency based on the shape and size of the bead and its surroundings (viscosity and bacterial load) [216]. The rotational frequency number is influenced by the total number of bacterial cells in a sample, and the MICs are calculated in the presence of an antibiotic [217].

Magnetic biosensors have found utility in detecting a wide array of AMR genes, encompassing those accountable for resistance to antibiotics like penicillin, tetracycline, and fluoroquinolones. This is exemplified by instances such as the identification of the *mecA* gene associated with MRSA and the *blaCTX-M* gene within *E. coli* strains.

Beads are also used by apparatuses based on the measurement of Brownian motion. In order to facilitate rapid AST and in situ bacterial monitoring, Wang et al. modified microbeads with vancomycin to form a self-powered sensor [218]. Vancomycin, a β-lactam antibiotic, shares structural similarities with the dd-transpeptidase enzyme and catalyzes the building of bacterial cell walls. Gram-negative bacteria have an additional layer of the outer membrane, but because of faults, d-Ala-d-Ala ligases are still susceptible to the environment [219]. As a result, vancomycin-modified microbeads can selectively trap bacteria over other types of cells or proteins and prevent the growth of their cell walls. Fluorescence microscopy is used to capture the Brownian motion due to the functionalized microbeads trapping and producing a change in diffusivity of bacteria. 

Piezoelectric/acoustic dynamic property measurements are possible with the quartz crystal microbalance (QCM). Using this technique, resonance frequency changes are caused by mass changes on the transducer surface. Due to its low cost and tolerance to chemical, thermal, and mechanical stresses, quartz is frequently used as the piezoelectric material for the transducer surface [220]. Various components like as antibodies, DNA or RNA capture probes, enzymes, or nanostructures, which possess sensitivity towards the target chemical, can be affixed onto the gold surface of the quartz crystal [204,221]. The resonance frequency of the cantilever, operating as a harmonic oscillator, undergoes alteration as a consequence of the interaction between the target species and the immobilized receptors situated on the cantilever. The cells adhere to the cantilever for assessing bacteria and their susceptibility or resistance, and the change in resonance frequency is noted as dependent on the extra mass and position [202,222,223]. The atomic force microscope (AFM) is one of the most crucial nanomechanical tools used in AST today since it can accurately detect ultrafine displacements [221]. Antibiotic exposure causes a rapid shift in the sensor’s fluctuations, which are produced by the bacteria [196].

#### 4.3.2. AST Optical Biosensors

AST optical biosensors employ a wide range of transducing techniques, including bio- and chemiluminescence, fluorescence, SPR, colorimetry, evanescent optical-plane waveguide, and reflectometric interference spectroscopy, among others [187,224,225]. Colorimetric sensors provide a variety of benefits, including a simple technique, lower cost, and the ability to observe the change of color with the ordinary eye or with basic equipment in quantitative analysis modes [226,227]. The use of chemical species, such as Resazurin, that change color in response to bacterial development [187,228] or the utilization of enzymes comprising enzymes like Horseradish Peroxidase (HRP) that alter substrates based on the number of microbial cells can aid real-time susceptibility measurements [226].

Dhar et al. [229] devised a biosensor that is capable of concurrently detecting point mutations in the *rpbB*, *katG*, and *gyrA* genes of MDR *Mycobacterium tuberculosis*. This is achieved through the utilization of split deoxyribozyme cascade probes.

#### Fourier-Transform Infrared (FTIR) Spectroscopy in AMR Diagnostics

Numerous biosensors rely on fluorescent labels, including nanoparticles [225,226,227,228,230,231]. Tang et al. [232] investigated the variation in pH resulting from the relatively fast buildup of metabolic products during cell development. In their work, a pH sensor for microfluidic was created by merging a chitosan hydrogel that is sensitive to pH with poly (dimethyl siloxane) (PDMS) microfluidic channels. To monitor the pH shift, Fourier transform–reflectometric interference spectroscopy (FT-RIFS) was utilized by injecting a suspension of bacterial cells treated with various antibiotics into a device and measuring the reflectance spectra. The real-time assessment of the rise in the effective optical thickness (EOT) was performed by measuring various antibiotics’ potential suppression of bacterial growth [196].

To date, technologies involving optical sensors and their applications in the health sciences and microbiological fields have demonstrated notable progress. Infrared (IR) spectroscopy and microscopy provide better spectral and spatial resolution, which facilitates the gathering of molecular-level biological information. FTIR spectroscopy permits the measurement of the IR light absorption by substrates such as lipids, carbohydrates, lipopolysaccharides, proteins, and nucleic acids, resulting in elaborate and deeply informative FTIR spectra [233]. The unique spectra of cell biomolecules offer a wealth of structural and functional information. IR spectroscopy has been used to differentiate the chemical changes associated with the development of AMR in prokaryotes [28]. In AMR research, FTIR has many advantages, including reliability, speed, cost-effectiveness, and environmental friendliness. However, the financial implication in handling the equipment potentially makes its deployment in settings with limited resources challenging, as a proof-of-concept AMR testing platform or for AST testing. Purification, culturing, and preparation of samples are required beforehand, and databases with spectra capable of differentiating the susceptibility of bacteria must exist as well [28]. The use of FTIR for AMR gene detection was reviewed by Kaprou et al. in 2021 [234]. Moreover, Salman et al. [235] utilized FTIR for the detection of the *E. coli* AMR gene. The fusion of FTIR microscopy and novel statistical classification techniques for spectral analysis has evolved into a potent approach that is capable of discerning molecular structural alterations linked to bacterial resistance against antibiotics. The computational classifier, grounded in infrared absorption spectra, is exceptionally attuned to delicate infrared spectral shifts that align with resistance-associated molecular modifications. The spectra from the study’s assessment of 496 distinct *E. coli* isolates likewise demonstrate similarities, unveiling subtle deviations in the form and intensity of diverse spectral attributes. Employing sophisticated multivariate and statistical strategies ensures a commendable classification level. These shifts enable the differentiation between antibiotic-resistant and sensitive *E. coli* isolates within mere minutes after initial cultivation [235]. This investigation serves as a proof-of-concept, highlighting the potential of this spectroscopic method in clinically managing bacterial infections. It achieves the characterization and classification of antibiotic resistance in considerably less time than conventional laboratory methods currently allow.

#### 4.3.3. AST Electrochemical Biosensors

For AST tests, electrochemical sensors have been suggested to be of great application [203,236,237]. Bacteria can display electroactivity and exhibit a vast array of redox reactions. Electrochemical sensors can detect the electrical reaction of microbial cells with a high degree of sensitivity, obviating the need for extensive isolation and culture and allowing measurements to be conducted immediately in biological and environmental matrices [203,238]. In the direct detection mode, the presence of membrane enzymes can either decrease or increase the transfer of electrons as the cell attaches itself to an antibody or bacteriophage immobilized on an electrode [239]. Meanwhile, in the indirect detection mode, the cell is induced to release measurable electroactive species [240]. Screen-printed electrodes (SPEs) provide a significant benefit compared to traditional electrochemical cell techniques by integrating all three electrodes (working, counter, and reference electrode) onto a single chip. This integration streamlines the analytical process, leading to reduced manufacturing costs and time [241,242,243]. In addition, electroanalytical methods may be utilized to greatly reduce susceptibility determination times for identifying pathogens [244,245,246]. Due to their great specificity and potential for identification, antibodies are an ideal complement to oligonucleotide-based capture probes for identifying entire cells [247]. Shi et al. deployed a biosensor that relies on the formation of a capture antibody–bacteria detection antibody sandwich immunological complex in order to provide a culture-independent approach for the rapid identification of BSIs [248]. This device utilizes redox electroactive enzymes combined with antibodies as sensing components. It consists of a three-electrode cell that has been modified with insulated gating electrodes (GE) to apply a gating voltage (VG) between the gating electrode and the working electrode. For this purpose, VG generates an electric field at the solution–enzyme–electrode interface, which modifies the interfacial charge distribution and thereby lowers the electron tunnel barrier. The first antibody that was specific to *E. coli* was used to immobilize on the surface of the working electrode, followed by incubating the sample with the biosensor. Using cyclic voltammetry (CV), the peak decrease of HRP was determined. *E. coli* bacteria were cultured with ampicillin in order to determine if the strains were sensitive or resistant to the antibiotic [196].

##### Aptamer

In biosensing and diagnostics, molecular recognition elements (MREs) have been studied extensively as potential antibody substitutes. Nucleic acid-based binding elements are among the most promising MREs because of their specificity and affinity. The Gold Laboratory published the first method known for screening an RNA-binding element [249]. Shortly thereafter, Szostak and Ellington discovered a new RNA binding element and named it “aptamer” [250]. Different bacterial components, including whole cells, polysaccharides, proteins, toxins, and spores, can function as binding sites for aptamers [251]. Aptamer application in medicines and diagnostics has grown significantly because of their versatility [252]. Single-stranded DNA aptamers have several advantages over their RNA counterparts. They exhibit various stem–loop structural variants continually and are firstly more stable than RNA aptamers [253,254].

The phrase “aptamer” comes from the Latin word *aptus*, which means “to fit,” and *meros*, which is a Greek word referring to “part.” Basically, aptamers are single-stranded nucleic acids consisting of up to 100 nucleotides, similar to RNA or DNA, and are considered to be relatively short. They usually create of loops, stems, and hairpins. Aptamers acquire a three-dimensional structure that enables them to attach to a specific target such as an antibody [252]. However, aptamers offer significant benefits over antibodies, making them an intriguing diagnostic and therapeutic option. Their chemical and physical stability gives aptamers an excellent advantage. Unlike proteins, which are regarded as denaturing at permanent and elevated temperatures, aptamers can revert to their original shape and retain their ability to attach to their intended target. Aptamers are also stable during long-term storage, making them appropriate for a variety of applications. In addition, both the identification and the manufacture of aptamers are less expensive. Animal vaccination is required for the discovery and synthesis of antibodies, followed by large-scale biotechnological fermentation for their recombinant manufacturing [255]. Additionally, there may be activity changes across different batches of a single antibody, necessitating extra testing of each batch. On the other hand, aptamers may be totally selected in vitro, even under non in vivo settings, allowing for the purpose-specific tailoring of the selected aptamer to the required conditions. The aptamer is then synthesized using well-defined chemical processes, making its manufacture extremely repeatable and reducing variances in their activity. In addition, this facilitates the alteration of aptamers during chemical synthesis. This alteration can only concern its stability, but fluorophores or other labels can also be added to enable the use of aptamers as extremely specific biosensors. Moreover, immunoglobulins often demonstrate a high degree of immunogenicity, which is an additional crucial factor. In comparison, aptamers are far less immunogenic and hazardous. Finally, the production of antibodies is restricted since they are generally acquired through animal vaccination. Therefore, isolating antibodies against hazardous targets or compounds that induce a strong immune response is not feasible at all. In addition, it is impossible to find antibodies against targets so tiny that the immune system cannot recognize them. Nevertheless, aptamers are chosen in vitro, rendering the toxicity or immunogenicity of the target unimportant, and they can even bind to extremely tiny targets, like ions, precisely [256].

**Figure 1:**
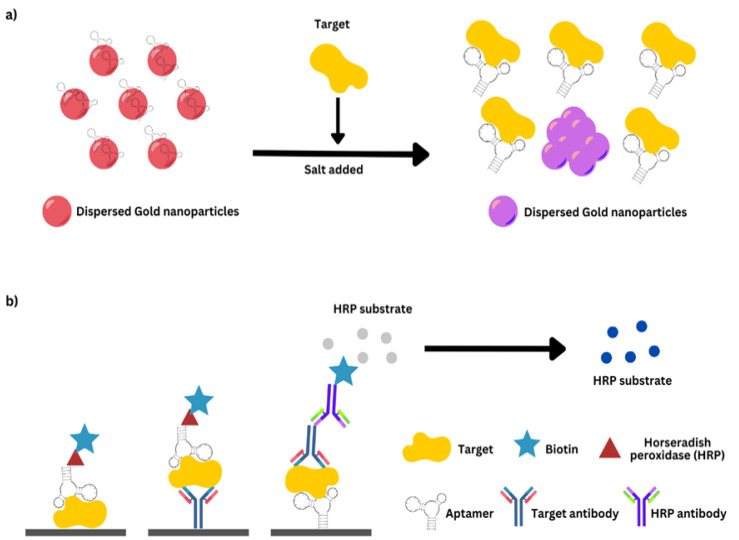
(**a**) An example describes a colorimetric sensor that detects red-to-purple transitions using gold nanoparticles (AuNPs). The left side of the diagram shows the first phase, where the aptamer coating evenly distributes AuNPs, turning them red. AuNPs aggregated after target–aptamer binding and salt addition. (**b**) An example of aptamer enzyme-linked sorbent assays is shown. The main antibody targets the molecule, while the secondary antibody can be used in different sandwich testing. (**a**,**b**) Visible color changes that may be measured with optical equipment.

To identify bacteria, aptamers have also been suggested in several studies [257,258,259,260]. Jo et al. [239] described the utilization of a capacitance sensor array that had been modified with aptamers to enable real-time monitoring of bacterial growth and drug sensitivity. As demonstrated in Figure 1, the surface of the Au-working electrode was immobilized with the strain-specific DNA aptamers. Bacteria can operate as capacitors linked in parallel between electrodes, yielding useful information about bacterial activity. The strain-specific DNA aptamers were used to immobilize the surface of the Au-working electrode. Bacteria can function as capacitors when connected in parallel between electrodes, providing useful data about the activity of the bacteria. When bacteria are exposed to inhibitory doses of antibiotics, capacitance decreases. The study can be finished in one hour [261]. Graphene field-effect transistors (G-FETs) are gaining popularity due to their high sensitivity in detecting biomarkers and DNA, scalability, biocompatibility, and ease of integration with a wide range of substrates [262,263].

Kumar and Wang [264] introduced a G-FET-based rapid, specific, and single-cell electrical device designed for the identification of antibiotic-resistant bacteria. Their approach, which aimed to detect pathogenic *Staphylococcus aureus* (*S. aureus*), involved the utilization of pyrene-conjugated peptides immobilized on G-FETs. Similarly, a comparable device was developed to differentiate between antibiotic-resistant and antibiotic-sensitive strains of *Acinetobacter baumannii*, underscoring the versatility of these devices in antibiotic-resistant bacteria identification. Notably, they harnessed electric-field-assisted binding to enhance bacterial adhesion, employing electrical pulses to coerce bacteria onto the graphene transducer.

Aptamer selection is often accomplished using a random process of systematic evolution of ligands by exponential enrichment (SELEX), which was originally described in 1990 [249,250]. In SELEX, the oligonucleotide pool undergoes repetitive incubation with the target, and the process is divided into four distinct phases: incubation, partitioning, recovery, and amplification. As a result, the affinity between the aptamer and the target gradually rises until the most specific aptamer is picked. The flexibility provided by SELEX’s binding conditions contributes to the adaptability of specific oligonucleotides to a wide range of cellular and non-cellular settings [265]. From 2016 to 2020, several publications detailed the diagnostic use of aptamers for bacterial illnesses. DNA aptamer biosensors were utilized in nearly all studies, showcasing their reliability for diagnostics, which outweighs the advantages of RNA’s three-dimensional capabilities. Only three of these investigations targeted resistant bacteria or ARG products.

In the initial study, Fan et al. [266] developed a peroxidase-like activity graphene-oxide aptasensor to detect the pure PBP2a protein, which is expressed by the *mecA* gene. Moreover, Maldonado et al. [267] designed a rapid label-free photonic pegylated aptasensor capable of identifying both the pure PBP2a protein and cultured MRSA-infected cells. Lastly, Qiao et al. [268] managed to detect PBP2a in *S. aureus* cells isolated from clinical plasma and nasal swab samples contaminated with MRSA strains, utilizing a single bacterial lysis step.

The aptamer-based diagnostic technique has mostly focused on the determination of the entire cell rather than its biologically distinct components, such as proteins or toxins. Consequently, owing to the well-recognized protein-SELEX technique, there remains a potential for the discovery of aptamers with exceptional specificity that can efficiently bind to proteins linked to ARGs, thereby enhancing diagnostic tools.

#### 4.3.4. Direct Fluorescent Imaging of Resistance Determinants by Fluorescence Resonance Energy Transfer (FRET)

The utilization of non-nucleotide probes labeled with reporter and quencher molecules, enabling fluorescence energy transfer (FRET), has been reported as a method for detecting enzyme resistance mechanisms, as demonstrated in the case of TEM-1 β-lactamases [269]. Focusing on β-lactamases, which are naturally occurring bacterial enzymes responsible for breaking down penicillin and cephalosporin antibiotics, offers distinct benefits. These enzymes are solely synthesized by antibiotic-resistant bacteria, ensuring labeling specificity with minimal disruption from native counterparts found in mammalian cells. The labile p-hydroxybenzylic esters, featuring FRET-quenched fluorescent tags, were linked to the Bla-sensitive cephalosporin structure. At this juncture, the sulfide bond underwent oxidation to sulfoxide to enhance stability. Following Bla hydrolysis, the liberated p-hydroxybenzylic derivatives were subjected to 1,6-elimination, resulting in the formation of fluorescent quinone–methide intermediates for targeted covalent labeling. In contrast with typical activity-based probes (ABPs) directly that interact with catalytic amino acid residues in the enzyme structure [248], the generation of quinone–methide nucleophilic traps mandates enzymatic cleavage of a precursor followed by subsequent spontaneous removal [270,271]. During these cascade reactions, the activated probes might not function as suicide inhibitors, thereby preserving enzyme activities to amplify fluorescent signals for observing single cells. This investigation employed three distinct fluorescent compounds—namely fluorescein, water-soluble Cy3, and near-infrared Cy5.5—each offering varied emission characteristics. This diversity facilitated microscopic imaging and high-sensitivity flow cytometry (HSFCM) analysis. The chosen fluorophores were initially quenched by DABCYL, BHQ2, and BHQ3 moieties, respectively, in order to optimize the signal-to-noise ratio. The labeling procedure coincided with the restoration of fluorescence, harnessed for the direct monitoring and screening of bacterial strains that exhibit resistance to antibiotics [272,273].

Following the enzymatic hydrolysis of probes to dissociate the quencher from the reporter, the resulting hydrolyzed probes serve as reactive electrophiles that interact with the resistance enzymes. Nevertheless, this approach has been exclusively utilized for TEM-1 β-lactamases in a preliminary investigation [269], and further comprehensive evaluation studies are required for its practical usefulness in routine microbiology diagnosis.

## 5. Future Perspectives and Emerging Technologies

### 5.1. Nanopore Sequencing

Nanopore sequencing, particularly using the de novo nanopore sequencer, has been extensively used in various applications related to bacterial assembly [274], the identification of viral pathogens [275], metagenomics, and the detection of ARGs [276,277]. The MinION nanopore sequencer was used to determine the structure and chromosomal location of a composite AMR in *Salmonella* Typhi [278]. It was also utilized to determine the location and structure of bacterial AMR determinants in an MDR strain of Enteroaggregative *E. coli* [279]. The utilization of a long-read analysis of WGS data facilitated the detection of mobile genomic elements harboring AMR determinants and unveiled the presence of several AMR determinants co-located on the same mobile element. As a result, this approach has improved our comprehension of the spread of co-located AMR determinants in MDR *E. coli* [279]. Schmidt et al. [256] showed that MinION was able to effectively identify bacterial infections and acquired resistance genes in urine samples without culturing within four hours [280]. This study emphasizes the significance of diagnostics based on whole-metagenome sequencing (WMS) and nanopore sequencing for selecting antimicrobial medication [280]. The Oxford Nanopore MinION long-read DNA sequencing instrument was utilized for the discovery of ARGs, the evaluation of their taxonomic origin, and the decoding of their genomic organization and potential association with mobilization markers [281]. Additionally, it has been used for the rapid identification of plasmids, phages, virulence markers, and ARGs in Shiga toxin-producing *E. coli* and for the quick detection of pathogens, plasmids, and ARGs in bacterial DNA isolated from positive blood cultures [282]. The MinION sequencer has also been evaluated for whole-genome production and the characterization of *Streptococcus suis*, predicting the MLST and detecting AMR profiles from genomes [283]. Overall, nanopore sequencing provides real-time data accessibility and can reveal the genetic underpinnings of AMR resistance, facilitating the comprehension of events leading to resistance acquisition.

### 5.2. Digital PCR

Digital PCR (dPCR) is the third generation of PCR technology, allowing for precise measurement by dividing the reaction into smaller partitions. dPCR stands out as a potent technique for nucleic acid quantification, offering distinct advantages compared to traditional PCR methods. The benefits of employing dPCR include heightened sensitivity, allowing for the detection of rare targets with remarkable precision by partitioning samples into numerous individual reactions. It provides absolute quantification of nucleic acids, eliminating the reliance on standard curves and, thus, enhancing result accuracy. Moreover, dPCR exhibits superior reproducibility and precision by being less susceptible to variations in reaction conditions and amplification efficiency. It reduces dependence on reference standards, which may be challenging to obtain for specific targets. This technique’s flexibility encompasses applications such as gene expression analysis, viral load quantification, and the detection of rare mutations, making it invaluable in both research and clinical contexts. Based on the techniques employed for segregating a reaction mixture, dPCR can be categorized into two distinct types: chip-based dPCR (cdPCR) and droplet-based dPCR (ddPCR).

As an example, Liu et al. [284] employed multiplexed droplet digital PCR (ddPCR) for the rapid and precise diagnosis of pediatric patients with suspected BSIs in China. The ddPCR panel effectively identified various bacteria, AMR genes (*blaKPC*, *mecA*, *OXA-48*, *NDM*, *IMF*, *vanA*, and *vanM*), and herpes family viruses, offering a comprehensive diagnostic solution. Among 76 enrolled pediatric patients, ddPCR showed a 47.9% positive rate, compared to 6.6% with blood culture, with a significantly shorter turnaround time (4.7 ± 0.9 h vs. 76.7 ± 10.4 h). ddPCR demonstrated 100% sensitivity and specificities ranging from 95.3% to 100.0%, identifying nine viruses.

The Integrated Comprehensive Droplet Digital Detection (IC3D) blood ddPCR platform integrates one-step blood droplet digital PCR with a high-throughput 3D particle counter for rapid, culture-free bacterial identification in BSIs. It offers high sensitivity, speed, and robustness by simplifying sample processing, using dPCR for precise quantification without internal references, and employing a high-throughput 3D particle counter. Key features include a streamlined one-step, blood inhibitor-resistant PCR, dPCR for single-cell-level quantification, and an efficient 3D particle counter for fast detection. IC3D technology effectively detected blaCTX-M-9 family ESBLs with a sensitivity of 10 CFU/mL within an hour from whole blood, making it versatile for antibiotic resistance gene detection and bacterial species identification in sepsis management [285]. Moreover, dPCR has the potential to be integrated with NGS techniques in order to accurately measure and analyze the obtained data. dPCR not only validates findings from NGS but also ensures the quality of sequencing data by addressing factors like adaptors, joint dimers, faulty link fragments, and lengthy fusion junctions.

Nonetheless, limitations persist. The lower limit is constrained by the sample volume in each microunit. The narrowing of the dynamic range arises from the limited number of microunits, and within these microunits, it is essential to recognize that not all DNA undergoes amplification, leading to continuous signals and inaccurate quantification at low levels. Also, dPCR is unsuitable for amplifying large amplicons and faces challenges related to low throughput in extraction and an elevated risk of sample contamination. Another problem with current dPCR systems is that they can identify only two colors, which makes it impossible to detect multiple targets within a single sample.

### 5.3. The Integration of CRISPR-Cas Systems with Aptamers

**Figure 2:**
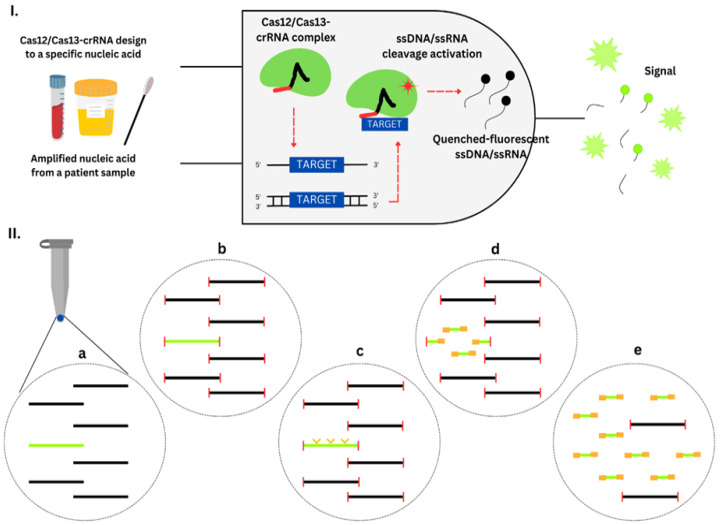
CRISPR-Cas-based diagnostics. (**I**) A logic gate type “AND” of CRISPR-Cas12/13-based diagnostics. Amplified nucleic acid from patient samples can be used as input. Attomolar RNA concentrations in serum or urine samples could be detected. Without purification, Cas13 could detect RNA in samples with serum concentration up to 2%. The diagnostic can use cfDNA liquid biopsy samples and DNA extracted from anal swabs. The diagnostic process also requires the Cas12/Cas13 system and a crRNA configuration that matches a target gene, such as an antibiotic resistance gene. The Cas12/Cas13-crRNA combination produces fluorescence output when it identifies target-positive material. The fluorescent signal is a result of a collateral trans-cleavage of the quenched-fluorescent ssDNA/ssRNA by Cas12/Cas13, respectively. (**II**) CRISPR-Cas9 enrichment for NGS detection. (**a**) DNA or cDNA sequences harboring the gene of interest are present (green). (**b**) DNA extremities are obstructed. (**c**) CRISPR-Cas9 system cleaves the target gene into fragments suitable for NGS (yellow arrows). (**d**) Ligation of universal sequencing adapters. (**e**) The target sequence is enriched, which is ready for sequencing.

CRISPR-Cas (clustered repetitive interspaced short palindromic repeats, CRISPR-associated enzymes) and aptamers technologies are gaining traction in the clinical detection and treatment of infectious diseases. Both may be used as an on-site diagnostic tool with a rapid TAT, making them more appealing than alternative approaches that target proteins or nucleic acids. CRISPR-Cas, a prokaryotic complex and ribonucleoprotein (RNP) is found in some proportion of bacteria and the majority of archaea, where it functions as an adaptive immune system for prokaryotes [286]. In three synchronized steps, the system provides protection against mobile genetic elements, including as bacteriophages, plasmids, and transposons: adaptation, CRISPR RNA (crRNA) synthesis, and interference [287]. As illustrated in Figure 2, most CRISPR-Cas systems rely on a clever technique to prevent self-targeting. This involves the detection of a short sequence referred to as the protospacer adjacent motif (PAM) during the adaptation and interference phases, which is unique to foreign nucleic acids [288,289]. As a benefit, these technologies provide a very specific detection of SNPs, which might be useful for distinguishing precisely any particular resistance gene variation [290]. Important constraints of the in vivo usage of aptamers include their vulnerability to nuclease breakdown and their quick removal owing to renal filtration; however, chemical modifications to the oligonucleotide structure have helped mitigate these drawbacks [291]. Considering the approval granted by the FDA, aptamer-based medicine currently exists, it is expected that the application of this technology to other disciplines, such as AMR, is imminent [292]. In contrast to therapy applications, a CRISPR-Cas-mediated diagnostic for identifying SARSCoV-2 was recently authorized by the FDA, opening the path for future uses. Its excellent scalability and multiplexing features are extremely beneficial for the identification and surveillance of the numerous ARG types. The target of either DNA or RNA, which validates the existence but not the functioning of ARGs, is a drawback of this method. Aptamers, in turn, target ARG products; however, a greater sensitivity, ideally to attomolar levels, may be necessary for independent bacterial detection in the bloodstream to be made feasible as the technique to be employed as a diagnostic tool [293]. In contrast to the more complicated and unpredictable process of aptamer selection, the point of flexibility of CRISPR-Cas, its easiness, and the prudent design of sgRNA/crRNA are advantageous. It is anticipated that CRISPR-Cas and aptamers can be coupled in the near future to treat and/or identify resistant bacterial diseases because of their complementary properties. Studies utilizing CRISPR-Cas and aptamers for diagnostics have proved their capacity to deliver TATs faster than the gold standard for AMR phenotypic assays [292].

### 5.4. Machine Learning and Predictive Analytics

Artificial intelligence (AI) is a field within the realms of science and engineering that focuses on the computational comprehension of intelligent behavior. There is no doubt that AI will be advantageous in numerous human professions, including clinical diagnosis and prognosis. AMR rates continue to grow; hence, it is crucial that institutions develop antibiotic stewardship programs to guarantee proper antibiotic usage, regulate antibiotics, and create AST antibiograms. When properly implemented, AI has the potential to revolutionize healthcare by speeding up the discovery of effective antibiotics, enhancing diagnostic precision, shortening the time between patient appointments, and reducing overall costs. Most proposed AI solutions for AMR are designed to complement rather than replace a doctor’s prescription or clinical judgment. They serve as valuable aids to simplify the healthcare professional’s work. In the context of infectious diseases, AI plays a transformative role in combating antibiotic resistance. Eventually, the use of prescribed antibiotics for the treatment of diseases necessitates reliance on data derived from local antibiotic stewardship programs. Such data have significant value in substantiating the prompt and efficacious treatment of bacteria, thereby mitigating the dissemination of resistant strains and invasive resistant bacterial variants [294].

Genotypic AMR prediction approaches require the sequencing of the organism, followed by the translation of the obtained sequence into a prediction using either a rule-based or a ML approach. Rule-based methods perform well in deeply studied organisms with a few highly penetrant resistance loci, such as *S. aureus* and *M. tuberculosis*, because they use previous knowledge to identify AMR genes (e.g., *mecA* and *vanA*) or resistance-causing mutations (e.g., *gyrA* S83L conferring fluoroquinolone resistance in *E. coli*). Rule-based approaches, due to their requirement for an in-depth understanding of the resistance mechanisms specific to each organism and involving manual curation, pose challenges when applied to a wide range of organisms and in accurately predicting complex AMR mechanisms, like polymyxin resistance in *K. pneumoniae*. Machine learning (ML) models have demonstrated an escalating ability to generate precise predictions of AST when trained on adequately extensive training datasets, even in the absence of prior knowledge regarding resistance mechanisms [295].

ML can be used to analyze huge datasets of microbial genetic information and clinical data to identify patterns and predict AMR. It can also help in developing personalized treatment plans based on individual patient characteristics. Predictive models of AMR, driven by ML, can act as a crucial link between specimen collection and outcomes derived from molecular and genotypic susceptibility analyses. These models expedite time-critical empirical antibiotic decisions.

Moran et al. [296] conducted an evaluation of the precision of an open-source ML algorithm (specifically XGBoost). The algorithm underwent training to predict antibiotic resistance in three Gram-negative bacterial species recovered from blood and urine samples of patients within 48 h of their arrival at the hospital [296]. The ML algorithm demonstrated a superior performance compared to both medical personnel and a basic risk assessment tool. Despite the potential limitation in generalizability, the authors advocated for the utility of a point-of-care decision support system that offers real-time, personalized therapeutic recommendations based on interim diagnosis and patient-specific risk factors. This is because the algorithm was exclusively trained to forecast resistance in specific cases and for particular antibiotics.

Garcia-Vidal et al. [297] utilized an ML technique by leveraging input data from the hospital’s electronic health records (EHRs). Their study focused on a cohort of hematological patients experiencing febrile neutropenia. The objective was to forecast which patients would require broad-spectrum coverage for MDR Gram-negative bacteria (MDR-GNB). This personalized antibiotic strategy aids in avoiding unnecessary antibiotic administration when not warranted [297]. The implementation of a Random Forest classifier trained on EHR data retrieved via a feature selection procedure could be employed to enhance the early detection of MDR infections in intensive care unit (ICU) patients. The model demonstrated an accuracy of 77% in this context [298].

Feretzakis et al. [299] conducted an evaluation of five ML algorithms with the aim of identifying indicators of antibiotic susceptibility. They utilized information collected from medical wards, such as patient demographics, culture, and antibiotic susceptibility test results. The researchers propose that clinicians could leverage ML insights derived from local antimicrobial susceptibility data to inform empirical treatment choices, with the most effective model achieving an accuracy of 75.8%. They also put forth a cost-efficient approach that is suitable for ICU environments, where concerns about AMR are prevalent. This approach involves utilizing a basic microbiology Laboratory Information System [299].

In the analysis of ICU AMR datasets, the multilayer perceptron and J48 algorithms exhibited a superior performance in terms of AUROC (0.726 and 0.724). The researchers found that by concentrating on specific MDR Gram-negative pathogens, the accuracy of the ML model was further enhanced (AUROC 0.933). This refinement aids in evidence-based decision making for empirical antibiotic selection [300].

Feature selection and ML techniques were used in a study conducted at a hospital affiliated with a Spanish university to identify antibiotic resistance in *Pseudomonas* bacteria [301]. The researchers concluded that ML algorithms such as LR, k-NN, DT, RF, and MLP contribute to streamlining clinical workflows. While performance metrics are provided, it is worth noting that, in most studies, there was no comparison between the performance of ML algorithms and the empirical clinical decisions made by physicians.

Beyond identifying AMR phenotypes, diverse ML modeling techniques have been harnessed by numerous scientists to forecast the antibiotic susceptibility patterns of pathogens. This enables the judicious selection of the most suitable treatment options. ML has shown promising results in predicting AMR. However, the accuracy of predictions is contingent upon several aspects, including the quality and quantity of data utilized, the selection of ML algorithm, and the features chosen for analysis. It is important to note that these studies were conducted on specific datasets and may not be generalizable to other datasets. Therefore, it is imperative to thoroughly assess the efficacy of ML models on new datasets before relying on them for clinical decision making.

## 6. Challenges and Opportunities

In line with earlier predictions, NGS will soon be a crucial component of routine diagnostics, eventually overtaking culture-based techniques as a precise and economical procedure in everyday clinical microbiology practice. This will need additional identification of specific virulence factors and sequence-based resistance testing, making culture unnecessary over the long term [302]. Due to its excellent resolution, high-throughput sequencing has the promising potential to enhance both diagnosis and patient treatment [303]. NGS can impact antibiotic stewardship by characterizing resistance based on the presence of a mechanism rather than only in terms of pharmacodynamics [304]. The vague relationship between genotype and phenotype is now a barrier, and there are many unresolved technological issues as well. Present hurdles include the imprecise connection between genotype and phenotype; moreover, a plethora of technical challenges have yet to be solved [304]. NGS, however, has the ability to substitute the diverse and challenging processes in a routine microbiological laboratory with a single, simple, and more successful technique as it becomes more affordable and straightforward to use [302].

Antibiotic susceptibility testing is fraught with challenges, including the rise of antibiotic-resistant bacteria, the absence of standardized testing approaches, and the protracted duration of testing procedures. Conversely, there are promising prospects on the horizon. These encompass the creation of novel technologies that facilitate swifter and more precise testing, harnessing big data for the scrutiny and prognostication of antibiotic resistance trends, and the establishment of antimicrobial stewardship initiatives that advocate judicious antibiotic utilization.

### 6.1. Standardization and Quality Control

AST and the significance of standardization and quality control cannot be overstated, as they are pivotal to ensure precise and dependable outcomes. Standardization pertains to the consistent adoption of methods and procedures, while quality control revolves around overseeing and confirming the accuracy and precision of the testing process. Internationally recognized organizations like the CLSI and the EUCAST play a critical role in establishing standards for AST. These organizations formulate guidelines and interpretive criteria that clinical laboratories employ to evaluate the susceptibility of bacterial isolates to various antibiotics. These guidelines consider factors such as the organism type, antibiotic used, and clinical significance of results. In addition to global entities, individual countries might institute their own standards and guidelines for AST. These standards often reflect international recommendations, while also considering localized epidemiological data and other region-specific factors. The standardization of AST guarantees the precision and reproducibility of results, which are vital for guiding accurate antibiotic therapy and the monitoring of antimicrobial resistance. Quality control mechanisms are equally crucial to ensure correct testing procedures and reliable results. Quality control forms a pivotal aspect of clinical laboratories, particularly in the realm of AST. External quality assessment programs are one avenue to implement quality control, involving the provision of samples with known susceptibility patterns for labs to assess their performance and identify areas for enhancement.

Adherence to standardized testing protocols is another fundamental facet of quality control. Laboratories should diligently follow established guidelines and protocols to maintain uniformity and accuracy in results. Regular maintenance and calibration of laboratory equipment, encompassing instruments like spectrophotometers and microscopes, and consistent monitoring of temperature and humidity levels are indispensable for quality control.

A robust error-monitoring and resolution system must be in place within laboratories. This might entail routine result reviews, the documentation of corrective measures taken, and continuous training and education for laboratory personnel. In essence, the interplay between standardization and quality control assures the integrity and reliability of AST outcomes, underpinning effective antibiotic treatment strategies and the vigilance against antimicrobial resistance. However, fresh validation of the diagnostic tools and standards are required for each release. As for the WGS, its introduction into the field of fast AST is still relatively recent. The greatest difficulty associated with WGS is bioinformatics, as broad databases are necessary for interpretating results [234]. Medical microbiology must develop a new generation of quick resistance testing assays to address the growing problem of a worldwide increase in illnesses caused by drug-resistant bacteria. The primary characteristics of these novel assays should be a considerable reduction in TAT and a high capacity for multiplexing [35].

Commercially produced kits are still undergoing developmental processes or need to fulfil a validation standard despite the market’s pressing expectations. There are a lot of obstacles that must be overcome. In the presence of high quantities of possible interferents, the detection of analytes at low concentrations is crucial. Enrichment and amplification are still necessary for analyzing real samples due to the requirement to identify target species in small quantities and in the presence of numerous other bacterial species. To that end, the integration of several technologies is required. By using nanoparticles or magnetic beads coated with ligands that target bacteria, the contaminating issue may be circumvented. In addition, developing new isothermal amplification methods will aid the biosensors sector. Recent research has focused on CRISPR-associated approaches for the detection of DNAs. This technique and other emerging biochemical methods are creative and potent instruments for the sensitive, quick, and selective monitoring of AMR genes. Transducers can be miniaturized with microfluidic platforms which will permit the use of multiplexed, compact, and user-friendly systems, resulting in the increased use of chemosensors and biosensors among end-users and industrial stakeholders. Research in chemo- and biomimetic receptors, such as peptides, imprinted polymers, and aptamers, will enhance selectivity and, therefore, bacterial detection, which is a crucial factor in improving the reliability of the biosensing method to control AMR. This necessitates the future creation of more effective sensing devices and biorecognition components to enable the precise identification of infections, particularly in medical situations [196]. Although various types of today’s technologies strive to fulfil the need for quick AST, none can be considered the best option. It is reasonable to expect that certain technologies may gain a significant market share in the field of rapid AST diagnosis in the future.

### 6.2. Access to Technologies in Resource-Limited Settings

Accessing AST technologies in resource-limited settings presents numerous challenges, including constrained financial resources, scarcity of trained personnel, inadequate laboratory infrastructure, and limited availability of antibiotics. Nonetheless, alternative approaches can be employed to address these challenges. One such alternative is the disk diffusion test, which offers simplicity and cost-effectiveness. This method enables the determination of bacterial susceptibility to antimicrobial agents. Similarly, the E-test, utilizing strips infused with antimicrobial agents, provides a quantitative approach to ascertain the MIC of bacteria. In resource-limited settings, the Microscopic Observation Drug Susceptibility (MODS) assay emerges as another viable option. This rapid and budget-friendly technique employs microscopy to identify bacterial growth in the presence of antimicrobial agents. Colorimetric assays represent additional alternatives, utilizing color changes to indicate bacterial growth. Molecular methods like PCR also prove valuable for detecting resistance genes.

Partnerships between developed and developing nations play a pivotal role in enhancing AST technology access in resource-limited contexts. Such collaborations can bolster funding for research and technology development, while furnishing training and capacity-building initiatives for healthcare professionals in developing countries. Expertise and technical support from developed countries can aid in the establishment of AST laboratories in these regions. These partnerships additionally contribute to ensuring affordability and accessibility of new technologies where they are most needed. The sharing of data and knowledge between developed and developing countries facilitates the development of effective strategies against antimicrobial resistance. This sharing extends to best practices for antimicrobial stewardship, infection control, and data on resistance patterns.

Point-of-care testing (POCT) stands out as a pivotal player in enhancing AST access within resource-limited settings. POCT facilitates swift and accurate infection diagnosis, guiding appropriate antibiotic utilization and reducing the risk of antimicrobial resistance. Furthermore, POCT allows for treatment response monitoring and adjustments, a critical function in settings where follow-up visits might be infrequent or challenging to coordinate. In essence, these alternative methods and partnerships hold the promise of bridging the gap in AST accessibility for resource-limited regions.

In the approaching years, chemosensors and biosensors are among the options for methodologies that are considered among the best with a promising outlook. The utilization of chemosensors and biosensors will lead to the creation of rapid POCTs with multiplexing capabilities. Progress in various transducing approaches will enable AMR monitoring in situations with limited resources and without the requirement for specially trained workers.

However, other costly and sophisticated diagnostic new methods will be difficult to access in countries with limited resources. Until sequence-based or MALDI-TOF MS methods become more economical and straightforward, rapid molecular techniques that are inexpensive and easy to perform, like FISH, may be a good option [34]. Microfluidics is a rapidly expanding discipline with vast promise and adaptability. In conjunction with tiny biosensing methods, numerous microfluidic technologies have considerable potential for the future. These microfluidic devices provide several benefits over traditional platforms, including minimum resource consumption, a cheap cost, simple operation, a shortened TAT, integration, automation, and mobility. Regarding microfluidic techniques, except for the upscaling of manufacturing technologies to enable mass production at a low cost, reaching a high degree of integration required for the pretreatment procedures, and improving the user-friendliness of the interface, there are no further challenges [234,305].

### 6.3. Regulatory and Policy Consideration

Antimicrobial susceptibility technologies, particularly those integrated into clinical practices, are subjected to rigorous regulatory scrutiny and approval processes. Regulatory bodies like the Food and Drug Administration (FDA) in the US and the European Medicines Agency (EMA) in the European Union are responsible for evaluating the performance, safety, and reliability of these technologies before permitting their marketing and usage within healthcare contexts. The regulatory requisites for AST exhibit variation contingent upon the country or region. For instance, the CLSI in the US furnishes guidelines for AST, while the FDA oversees the regulation of AST systems utilized in clinical settings. In parallel, the EUCAST extends guidelines for AST within the European Union.

Adhering to these guidelines is pivotal to ensure the precision and dependability of outcomes. Furthermore, specific regulations or policies might be in place for the deployment of AST technologies across various healthcare environments, encompassing hospitals and long-term care facilities. The landscape of antimicrobial stewardship also exerts a significant influence on the utilization of AST technologies. These strategies are tailored to foster prudent antibiotic use and mitigate the emergence of antibiotic-resistant bacteria.

An avenue through which policies impact susceptibility technologies involves the imposition of antimicrobial stewardship programs within healthcare facilities. These programs commonly integrate guidelines for judicious antibiotic usage and mandates for monitoring and reporting antibiotic administration and resistance trends. Additionally, policies can exert influence on the development and approval of novel susceptibility technologies. Regulatory agencies might necessitate evidence of clinical utility and discernible contributions to antimicrobial stewardship before granting approval for these technologies in healthcare settings.

AMR constitutes a worldwide health hazard that demands global cooperation. Regulatory and policy structures can play a role in fostering international data sharing and collaboration among nations, thereby enhancing global AMR surveillance and response initiatives. This collective approach holds particular significance for antimicrobial susceptibility technologies, as it facilitates the amalgamation of resources, insights, and proficiencies. Consequently, this pooling of assets fosters the generation of more precise and all-encompassing data, while also facilitating the creation of enhanced tools and tactics to counteract antimicrobial resistance. The AMR crisis necessitates a concerted effort from scholars, those managing and accessing risk, the government, and the private sector to improve the present techniques for diagnosis and treatment through the development of novel tools that circumvent the shortcoming and limitations of the gold standards and existing AST methods.

## 7. Conclusions

The primary drawbacks of the present technologies include the requirement for sample pretreatment processes; limited sensitivity; the inability to identify microorganisms in some instances; and the absence of integration, automation, and portability. Regarding the first three elements, to detect a number of infections, extensive biological processes (culture, isolation, and identification) are necessary. To expedite the approval and commercialization of new testing platforms with superior performance characteristics, it is of the utmost importance to prioritize and strive for considerable progress in their development. Equally imperative is investing time and effort in the enhancement of current methodologies, technologies, and platforms. In conclusion, with the challenges and threats AMR poses, developing dependable, sensitive, and cost-effective diagnostics will aid in the fight against AMR. The implementation of rapid diagnostic technologies, particularly within primary care settings, has the potential to enhance and facilitate the effectiveness and specificity of therapeutic interventions. In addition, enhanced monitoring technologies, such as mobile apps and surveillance programs, are required to monitor antimicrobial usage. The integration of machine learning and data mining techniques, together with automation, is anticipated to have a pivotal impact on the next age of diagnostic practices. Epidemiological surveillance is of utmost relevance in addressing AMR, as it provides vital information for the development and oversight of treatment recommendations, antibiotic stewardship programs, and public health interventions, as well as the advancement of novel antimicrobials and vaccines [306]. The development of cutting-edge methods and technology to combat AMR and AST, along with surveillance programs enabling enhanced and streamlined data transfer, would significantly contribute to limiting the negative impacts of the AMR threat [28].

## Data Availability

Not applicable.
